# Analysis of lactate metabolism-related genes and their association with immune infiltration in septic shock via bioinformatics method

**DOI:** 10.3389/fgene.2023.1223243

**Published:** 2023-07-26

**Authors:** Huimin Jiang, Yun Ren, Jiale Yu, Sheng Hu, Jihui Zhang

**Affiliations:** ^1^ Emergency Intensive Care Unit, Ningxiang People’s Hospital Affiliated to Hunan University of Chinese Medicine, Changsha, China; ^2^ Emergency Department, Ningxiang People’s Hospital Affiliated to Hunan University of Chinese Medicine, Changsha, China

**Keywords:** diagnosis of septic shock, hypoxia, lactate metabolism-related genes, immune infiltration, inflammation response

## Abstract

**Background:** Lactate, as an essential clinical evaluation index of septic shock, is crucial in the incidence and progression of septic shock. This study aims to investigate the differential expression, regulatory relationship, clinical diagnostic efficacy, and immune infiltration of lactate metabolism-related genes (LMGs) in septic shock.

**Methods:** Two sepsis shock datasets (GSE26440 and GSE131761) were screened from the GEO database, and the common differentially expressed genes (DEGs) of the two datasets were screened out. LMGs were selected from the GeneCards database, and lactate metabolism-related DEGs (LMDEGs) were determined by integrating DEGs and LMGs. Protein-protein interaction networks, mRNA-miRNA, mRNA-RBP, and mRNA-TF interaction networks were constructed using STRING, miRDB, ENCORI, and CHIPBase databases, respectively. Receiver operating characteristic (ROC) curves were constructed for each of the LMDEGs to evaluate the diagnostic efficacy of the expression changes in relation to septic shock. Finally, immune infiltration analysis was performed using ssGSEA and CIBERSORT.

**Results:** This study identified 10 LMDEGs, including *LDHB, STAT3, LDHA, GSR, FOXM1, PDP1, GCDH, GCKR, ABCC1*, and *CDKN3*. Enrichment analysis revealed that DEGs were significantly enriched in pathways such as pyruvate metabolism, hypoxia pathway, and immune-inflammatory pathways. PPI networks based on LMDEGs, as well as 148 pairs of mRNA-miRNA interactions, 243 pairs of mRNA-RBP interactions, and 119 pairs of mRNA-TF interactions were established. ROC curves of eight LMDEGs (*LDHA, GSR, STAT3, CDKN3, FOXM1, GCKR, PDP1*, and *LDHB*) with consistent expression patterns in two datasets had an area under the curve (AUC) ranging from 0.662 to 0.889. The results of ssGSEA and CIBERSORT both showed significant differences in the infiltration of various immune cells, including CD8 T cells, T regulatory cells, and natural killer cells, and LMDEGs such as *STAT3, LDHB, LDHA, PDP1, GSR, FOXM1*, and *CDKN3* were significantly associated with various immune cells.

**Conclusion:** The LMDEGs are significantly associated with the immune-inflammatory response in septic shock and have a certain diagnostic accuracy for septic shock.

## 1 Introduction

Sepsis is life-threatening and caused 19.7% of all deaths across the world in 2017 (Rudd et al., 2020). When sepsis causes systemic vasodilation and subsequent tissue hypoperfusion, septic shock occurs, which is characterized by hypotension and tissue hypoperfusion (Singer et al., 2016; Kanjee et al., 2023). Septic shock is the direct cause of death in sepsis, and once it occurs, the short-term mortality rate can be as high as 52% (Pavon et al., 2013). The new definition of sepsis emphasizes that instead of the infection itself, dysregulated host response to the infection is what drives the development of disease (Singer et al., 2016). However, many drugs aimed at blocking inflammation and regulating immunity that have been proposed based on this definition have not been observed to have the expected therapeutic benefits in clinical studies (van der Poll et al., 2017). Therefore, exploring the mechanisms of sepsis progression to shock from other perspectives and identifying potential therapeutic targets may be of great significance in reducing its mortality rate.

As is widely known, in patients suffering from sepsis, especially septic shock, lactate levels are often significantly elevated and closely related to the severity of the condition and the prognosis (Casserly et al., 2015). In fact, in the progression of sepsis, blood lactate serves not only as a marker of organ perfusion and tissue hypoxia, but also participates in the progression of sepsis to shock and death through multiple signaling pathways, such as regulating energy metabolism and immune inflammatory responses (Brooks et al., 2022). An animal experiment demonstrated that intraperitoneal injection of lactate to mice with lactate clearance impairment directly resulted in their death, whereas intraperitoneal injection of the maximum tolerated dose of lactate to normal mice did not (Vandewalle et al., 2021). It is evident that the accumulation of lactate in the body can directly cause organ dysfunction and even death. On the one hand, lactate can decrease effective circulating blood volume and aggravate organ dysfunction by dilating blood vessels and altering vascular permeability, such as by increasing vascular permeability through lactate receptors GPR81 or targeting VE-cadherin (Yang et al., 2022a; Yang et al., 2022b). On the other hand, lactate participates in essential steps of immune cell energy metabolism, differentiation, migration, and the release of inflammatory factors in the uncontrolled inflammation response of sepsis. During the excessive immune response period of sepsis, in order to solve the problem of enormous energy demand, the vast majority of immune cells switch from oxidative phosphorylation to glycolysis and produce lactate through metabolic reprogramming, despite the low efficiency of glycolysis compared to oxidative phosphorylation (Ye et al., 2022). However, the accumulation of lactate can in turn inhibit the glycolysis of monocytes (Dietl et al., 2010). Lactate can induce the differentiation direction of naive CD4^+^ T cells and increase the percentage of Treg cells (Comito et al., 2019), regulate the polarization of macrophage M2 (Colegio et al., 2014), and also limit antigen presentation and migration of dendritic cells (Puig-Kroger et al., 2003; Gottfried et al., 2006). Through GPR81-mediated YAP inactivation, lactate can prevent the synthesis of cytokines that promote inflammation in macrophages (Brand et al., 2016), and high levels of lactate additionally reduce the synthesis of cytokines in NK and T cells and cause immune cell death (Husain et al., 2013; Brand et al., 2016; Long et al., 2018).

Given the significant function of lactate in the onset and progression of septic shock, this research aims to use bioinformatics methods to identify lactate metabolism-related differentially expressed genes (LMDEGs) from datasets related to septic shock. The study will analyze the functional signaling pathways and regulatory networks enriched by these genes, their diagnostic efficacy, and the relationship with immune infiltration.

## 2 Methods

### 2.1 Data download

The R package GEOquery (Davis and Meltzer, 2007) was utilized to download two *Homo sapiens* data sets, GSE26440 (Wong, 2012) and GSE131761 (Martinez-Paz et al., 2021), from the GEO database (Barrett et al., 2007). The GSE26440 data set (platform: GPL570 [HG-U133_Plus_2] Affymetrix Human Genome U133 Plus 2.0 Array) comprised 130 whole blood samples, of which 98 were from septic shock patients and 32 from healthy people. The GSE131761 data set (platform: GPL13497 Agilent-026652 Whole Human Genome Microarray 4x44K v2) was composed of 129 whole blood samples, including 81 septic shock patients, 33 patients in shock caused by other factors, and 15 healthy people.

The GPL files were used to annotate all the probe sets. In the subsequent analysis, all expression profile data samples in GSE26440, consisting of 98 whole blood samples from patients with septic shock (septic shock group) and 32 whole blood samples from matched normal controls (control group), were included. Additionally, in GSE131761, the whole blood expression profile data samples of patients, who were experiencing septic shock or other types of shock were also included in the following analysis. This set comprised 81 whole blood samples from patients with septic shock (septic shock group) and 33 matched whole blood samples from non-septic shock patients (control group). In septic shock, lactate may not solely be a consequence of circulatory dysfunction but could potentially serve as a causative factor, actively participating in the dysregulated immune-inflammatory response during some early stage of sepsis, thus promoting the onset of shock. Conversely, in other types of shock present in this dataset, such as cardiogenic shock, lactate is more likely to be a consequence of inadequate effective circulating volume. Therefore, utilizing this dataset to compare the differential gene regulation of lactate production and clearance between septic shock and non-septic shock patients would be more advantageous in eliminating the overlapped lactate-regulating genes due to circulation dysfunction during the shock phase. With the exception of the 15 healthy individual samples that were excluded from the dataset GSE131761, all gene expression levels encompassing both datasets were incorporated in the present investigation, thereby precluding any instances of data omission. More thorough specifics about the data sets are offered in Table 1.

**TABLE 1:** Septic shock data set information list.

	GSE26440	GSE131761
Platform	GPL570	GPL13497
Species	*Homo sapiens*	*Homo sapiens*
Tissue	Whole blood	Whole blood
Samples in septic shock group	98	81
Samples in Control group	32	33
Time of blood samples obtained	The first 24 h of admission to intensive care unit	Within 24 h of shock diagnosis
Reference	Identification of pediatric septic shock subclasses based on genome-wide expression profiling	Distinguishing septic shock from non-septic shock in postsurgical patients using gene expression

Additionally, we gathered lactate metabolism-related genes (LMGs) connected to lactate metabolism via GeneCards. Moreover, we obtained LMGs from the GeneCards (https://www.genecards.org/), which provides detailed information on human genes (https://www.genecards.org/) (Stelzer et al., 2016). We searched for “Lactate Metabolism” as the keyword and filtered for “Protein Coding” LMGs to obtain 50 LMGs, as presented in Table 2.

**TABLE 2:** Lactate Metabolism genes list.

Lactate metabolism genes list		
LDHB	HCAR1	GCDH
LDHA	STAT3	HK2
INS	SOD1	G6PC1
SLC16A1	KRAS	HTT
SLC16A4	TNF	FOXM1
PIK3C2A	IL6	GPR68
MMP2	ABCC1	MB
PDP1	ADRB2	CDKN3
NPPA	GSR	GCKR
DISC1	P4HB	PTP4A2
LDHD	CAV1	ATOX1
TGFB2	DNM1L	SHC3
F2	HIF1A	MACC1
CS	IL1B	PPP1R3B
SPAG6	AKR1B1	
BSG	FOXO3	
PDYN	KCNJ5	
GPT	PC	

### 2.2 Identification of LMDEGs related to septic shock

To identify differentially expressed genes (DEGs) associated with septic shock, we employed the limma package to normalize the data sets GSE26440 and GSE131761. Subsequently, limma package was utilized to perform differential analysis on the datasets GSE26440 and GSE131761 to determine DEGs between various groups. We set *|LogFC|* >0 and *P.adj* < 0.05 as the threshold for selecting DEGs for further investigation. Genes exhibiting *logFC* > 0 and *P.adj* < 0.05 were classified as upregulated, whereas those with *logFC* < 0 and *P.adj* < 0.05 were designated as downregulated.

To identify lactate metabolism-related differentially expressed genes (LMDEGs) in septic shock, we performed several steps. Firstly, we obtained DEGs with *|logFC|* > 0 and *P.adj* < 0.05 from the GSE26440 and GSE131761 datasets. Then, to find the common DEGs, we created a Venn diagram using the intersection of these DEGs from both datasets. Next, we intersected the common DEGs with the LMGs obtained from the GeneCards database to obtain the LMDEGs, which were also represented in a Venn diagram. The R package ggplot2 for the volcanic map and pheatmap for the heat map were chose to present the outcomes of various analyses.

### 2.3 Gene ontology (GO) and Kyoto Encyclopedia of Genes and Genomes (KEGG) enrichment analysis of LMDEGs

R package clusterProfiler was utilized to perform KEGG and GO (Yu et al., 2012), We considered *P.adj* < 0.05 and FDR values (*q.value*) < 0.05 to be statistically significant entry screening criteria. The Benjamin Hochberg method was used for *p*-value correction.

### 2.4 Gene set enrichment analysis (GSEA)

In this study, we employed GSEA to investigate the contribution of predefined gene sets to the phenotype by assessing the distribution trend of genes (Subramanian et al., 2005). The following GSEA parameters were chosen: Benjamini–Hochberg *p*-value correction, a seed of 2020, a calculation number of 1000, a minimum of 10 genes per gene set, a maximum of 500 genes, and a minimum of 10 genes per gene set. From the MSigDB (Liberzon et al., 2015), we obtained the c2.cp.all.v2022.1.Hs.symbols.gmt [All Canonical Pathways] (3050) gene set. We used *P.adj* < 0.05 and *FDR* value < 0.05 as screening criteria for significant enrichment.

### 2.5 Protein-protein interaction (PPI)

Numerous biological functions, including signal transmission, gene control, metabolism, and cell cycle regulation, rely on PPI networks. The STRING database is a comprehensive resource for investigating known and predicted PPIs (Szklarczyk et al., 2019). In this study, we employed the STRING database to construct a PPI network with a minimum required interaction score of low confidence (0.150) and visualised the PPI network model using Cytoscape version 3.9.1.

### 2.6 Construction of mRNA-miRNA, mRNA-RNA binding protein (RBP), mRNA-transcription factors (TF) interaction networks

The miRDB database is a tool for predicting miRNA target genes and providing functional annotations (Chen and Wang, 2020). In this study, we utilized the miRDB database to predict miRNAs that interact with LMDEGs. We then identified mRNA-miRNA interaction pairs with a Target Score ≥80 from the miRDB database interaction network.

The ENCORI database (version 3.0) is a comprehensive platform that provides diverse visual interfaces for exploring miRNA targets, based on data mining of CLIP-seq and degradome sequencing (for plants) (Li et al., 2014). In this study, the mRNA-RBP interaction network was built using the ENCORI database to predict the RNA-RBP interactions with LMDEGs.

Millions of transcription factors and genes can be predicted to have transcriptional regulatory interactions using the CHIPBase database, version 3.0 (https://rna.sysu.edu.cn/chipbase/) (Zhou et al., 2017). Additionally, the hTFtarget database (http://bioinfo.life.hust.edu.cn/hTFtarget) is a comprehensive database with information on human TFs and their associated regulating targets (Zhang et al., 2020). In this study, we utilized the above-mentioned two databases to identify TFs that bind to LMDEGs. The resulting data were visualized using Cytoscape software.

### 2.7 Receiver operating characteristic curve (ROC)

ROC is a coordinate graph analysis tool that can be used to select the best model, discard suboptimal models, or set optimal thresholds within the same model (Mandrekar, 2010). By using the construction method, ROC curves reflect the relationship between sensitivity and specificity of continuous variables. The area under the curve (AUC) ranges between 0.5 and 1, where a higher AUC signifies better diagnostic performance. To evaluate the diagnostic effectiveness of LMDEGs on septic shock, we drew the ROC using the R package “proc” and calculated the AUC for the GSE26440 and GSE131761 datasets.

### 2.8 Immune infiltration analysis

The single-sample gene-set enrichment analysis (ssGSEA), employing 28 gene sets from published literature to identify various human immune cell subtypes is a well-established method for identifying and quantifying tumour-infiltrating immune cell subtypes in the tumour microenvironment (Bindea et al., 2013). In this study, we utilized the ssGSEA algorithm, available in the R package GSVA, to compute the enrichment scores, which represent the infiltration levels of different immune cell types in each sample. We displayed the differences in immune infiltration by box plots and computed the correlation between immune cells and LMDEGs using gene expression matrices from GSE26440 and GSE131761. Furthermore, we generated a correlation heatmap plot using the R package pheatmap to visualize the relationship between immune cells and LMDEGs.

CIBERSORT is an algorithm for immune infiltration analysis that uses linear support vector regression to deconvolve transcriptional expression matrices, enabling estimate of immune cell population composition and abundance in mixed cell populations (Newman et al., 2015). We uploaded the whole blood sample matrix data from the septic shock and control groups in the GSE26440 and GSE131761 datasets to CIBERSORT. The differences in immune cell infiltration abundance in GSE26440 and GSE131761 were presented through stacked plots. We calculated Spearman correlation coefficients using the immune cell infiltration levels within different groups and visualized the results using the R package ggplot2. Then, we calculated the correlation between immune cells and LMDEGs using the gene expression matrices from GSE26440 and GSE131761 and displayed the results using a heatmap plot generated by the R package pheatmap.

### 2.9 Statistical analysis

All data processing and analysis in this study were performed using R (Version 4.1.2). Continuous variables were reported as mean ± standard deviation. For comparing two groups, Wilcoxon rank sum tests were used, and Kruskal–Wallis tests were used for comparing three or more groups. The chi-square or Fisher’s exact test was employed to examine the statistical significance between two categorical variables. Unless otherwise indicated, Spearman correlation analysis was performed to calculate the correlation coefficient between different molecules, and all results were considered statistically significant when the *p*-value was less than 0.05.

**FIGURE 1:**
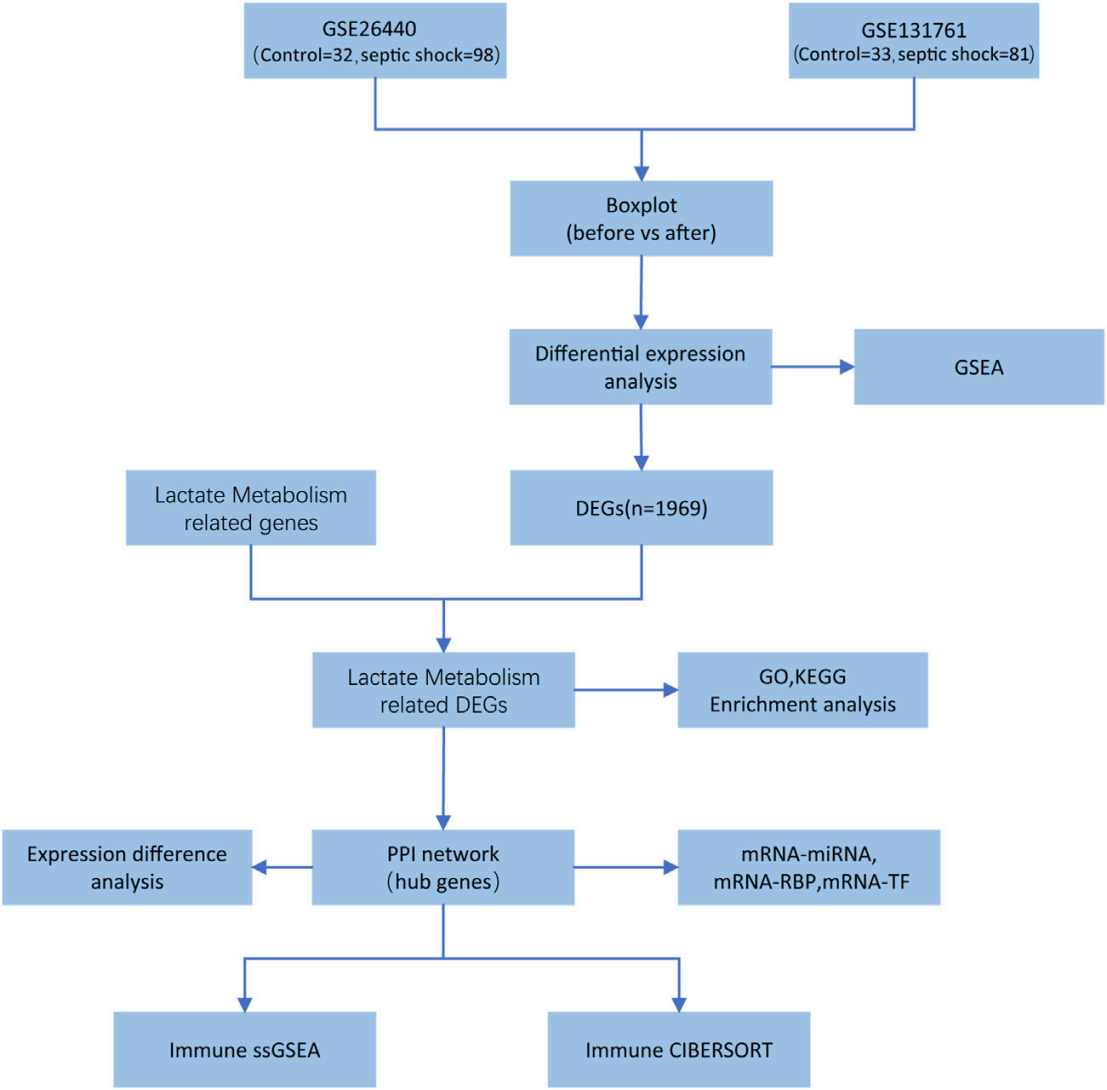
Flowchart of data analysis.

The entire research process is depicted in Figure 1.

## 3 Results

### 3.1 Identification of LMDEGs

**FIGURE 2:**
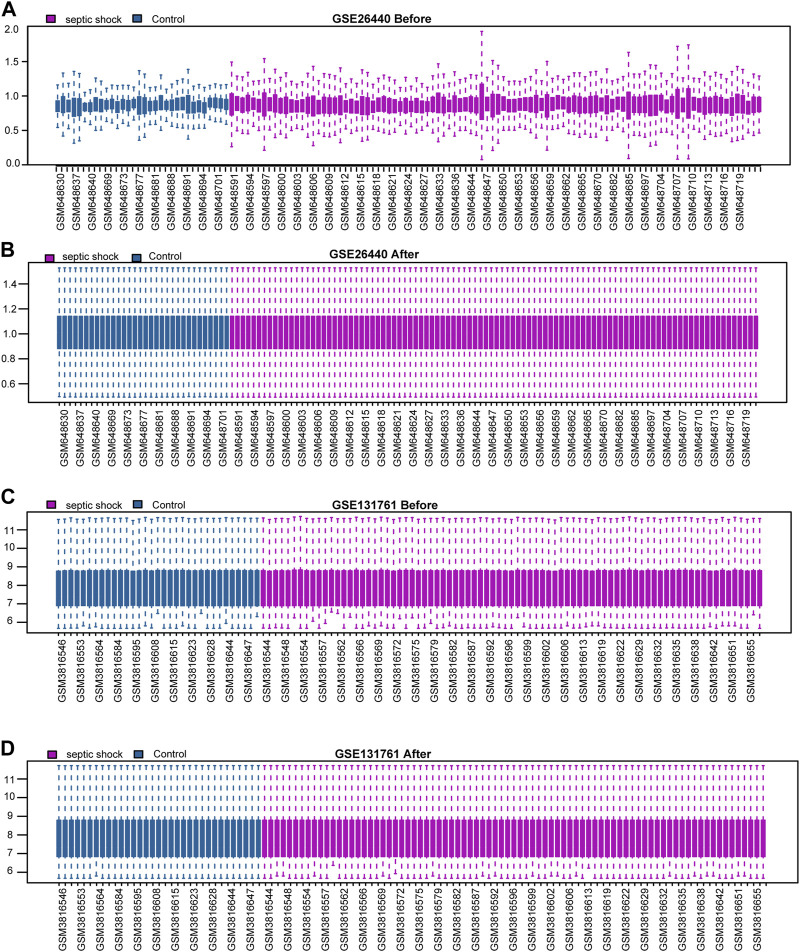
Standardization processing of septic shock datasets. **(A,B)** Boxplots of the GSE26440 dataset before **(A)** and after **(B)** normalization. **(C,D)** Boxplots of the GSE131761 dataset before **(C)** and after **(D)** normalization.

We initially normalised two septic shock datasets, GSE26440 and GSE131761, using the R package limma. The GSE26440 dataset comprised of 130 samples, including 98 septic shock samples (labelled as “Septic shock”) and 32 normal samples (labelled as “Control”) (Figures 2A, B). Likewise, the GSE131761 dataset comprised of 114 samples, with 81 septic shock samples (labelled as “Septic shock”) and 33 other types of shock samples (labelled as “Control”) (Figures 2C, D). As illustrated in Figures 2A–D, after normalisation, the batch effects between samples in both septic shock datasets were substantially mitigated, and the expression profiles of the samples became more consistent.

**FIGURE 3:**
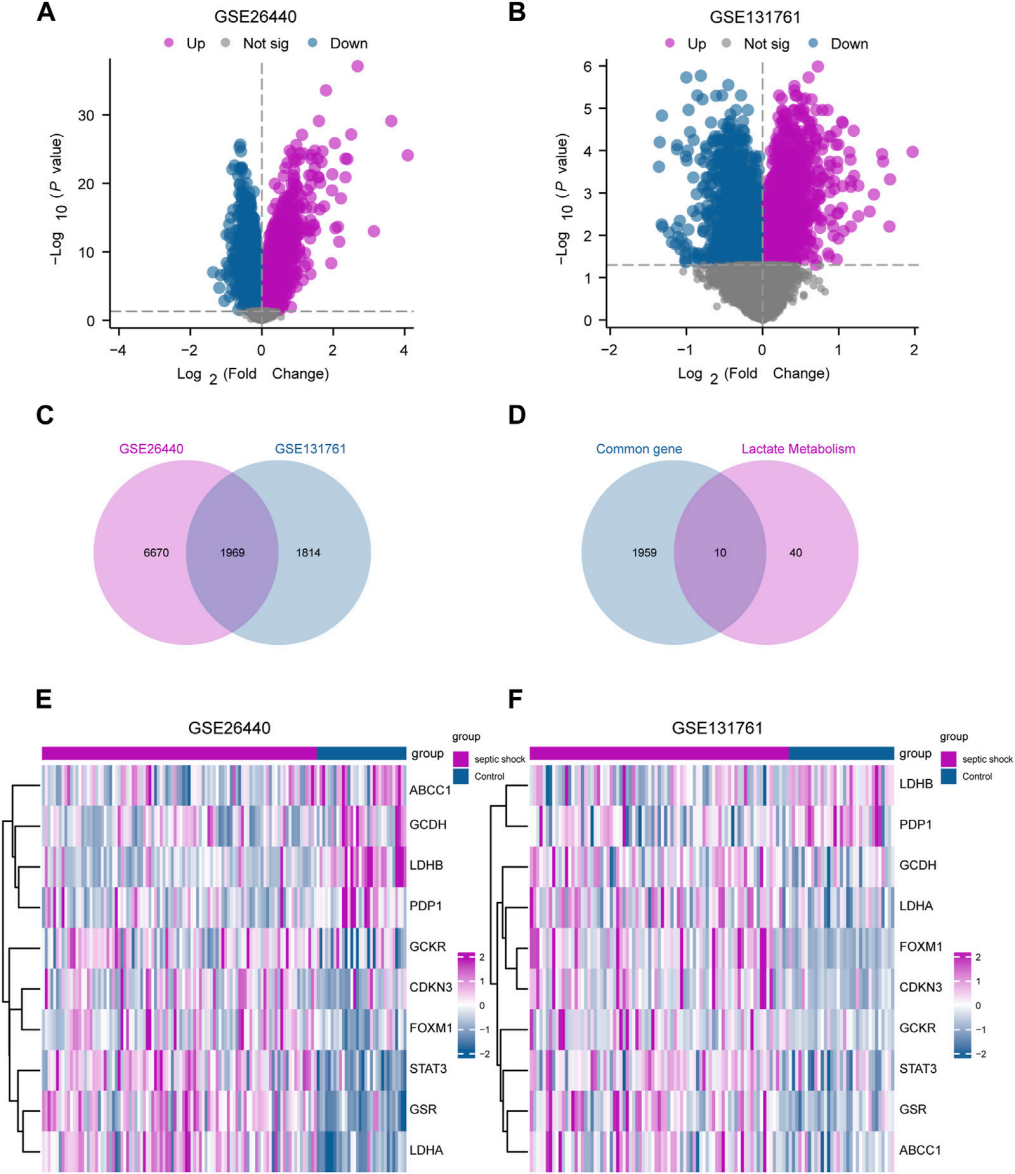
Identification of LMDEGs from septic-shock datasets. **(A)** Volcano plot for DEGs in GSE26440. **(B)** Volcano plot for DEGs in GSE131761. **(C)** Venn diagram of DEGs from GSE26440 and GSE131761. **(D)** Venn diagram of common DEGs and LMGs in the datasets. **(E,F)** Heatmap of LMDEGs in GSE26440 **(E)** and GSE131761 **(F)** LMDEGs: lactate metabolism-related differentially expressed genes; DEGs: differentially expressed genes; LMGs: lactate metabolism-related genes.

In this study, limma package in R was utilized to conduct differential gene expression analysis on the GSE26440 and GSE131761 datasets, aiming to identify DEGs between the septic shock group and the control group. In the GSE26440 dataset, consisting of 130 samples, 21,652 DEGs were identified, with 8,639 genes meeting the criteria of *|logFC|* > 0 and *P.adj* < 0.05. Among these genes, 4,799 were upregulated (*logFC* > 0), and 3,840 were downregulated (*logFC* < 0) (Figure 3A). We generated a volcano plot to visualize the DEGs. Similarly, in the GSE131761 dataset, consisting of 114 samples, 21,752 DEGs were identified, with 3,783 genes meeting the criteria of *|logFC|* > 0 and *P.adj* < 0.05. Among these genes, 1,678 were upregulated (*logFC* > 0), and 2,105 were downregulated (*logFC* < 0), and a volcano plot was plotted to visualize the DEGs (Figure 3B). By taking the intersection of all the DEGs with *|logFC|* > 0 and *P.adj* < 0.05 from both datasets, we obtained a list of 1,969 common DEGs (Figure 3C). We further intersected these common DEGs with LMGs, resulting in the identification of 10 LMDEGs (Figure 3D), including *lactate dehydrogenase B (LDHB)*, *signal transducer and activator of transcription 3 (STAT3)*, *lactate dehydrogenase A (LDHA)*, *glutathione-disulfide reductase (GSR)*, *forkhead box protein M1 (FOXM1)*, *pyruvate dehyrogenase phosphatase catalytic subunit 1 (PDP1)*, *glutaryl-CoA dehydrogenase (GCDH)*, *glucokinase regulatory protein (GCKR)*, *ATP-binding cassette subfamily C member 1 (ABCC1)*, and *cyclin-dependent kinase inhibitor 3 (CDKN3)*. Heatmaps were generated to visualize the expression differences of these genes in the septic shock group and control group in both datasets (Figures 3E, F). Among the 10 LMDEGs, *LDHA*, *GSR, STAT3, CDKN3, FOXM1,* and *GCKR* were upregulated (*logFC* > 0) in septic shock groups of both datasets, while *PDP1* and *LDHB* were downregulated (*logFC* < 0) in septic shock groups of both datasets. *ABCC1* and *GCDH* were downregulated (*logFC* < 0) in septic shock group of the GSE26440 dataset, but upregulated (*logFC* > 0) in septic shock group of the GSE131761 dataset.

### 3.2 Functional and pathway enrichment analysis of LMDEGs

**FIGURE 4:**
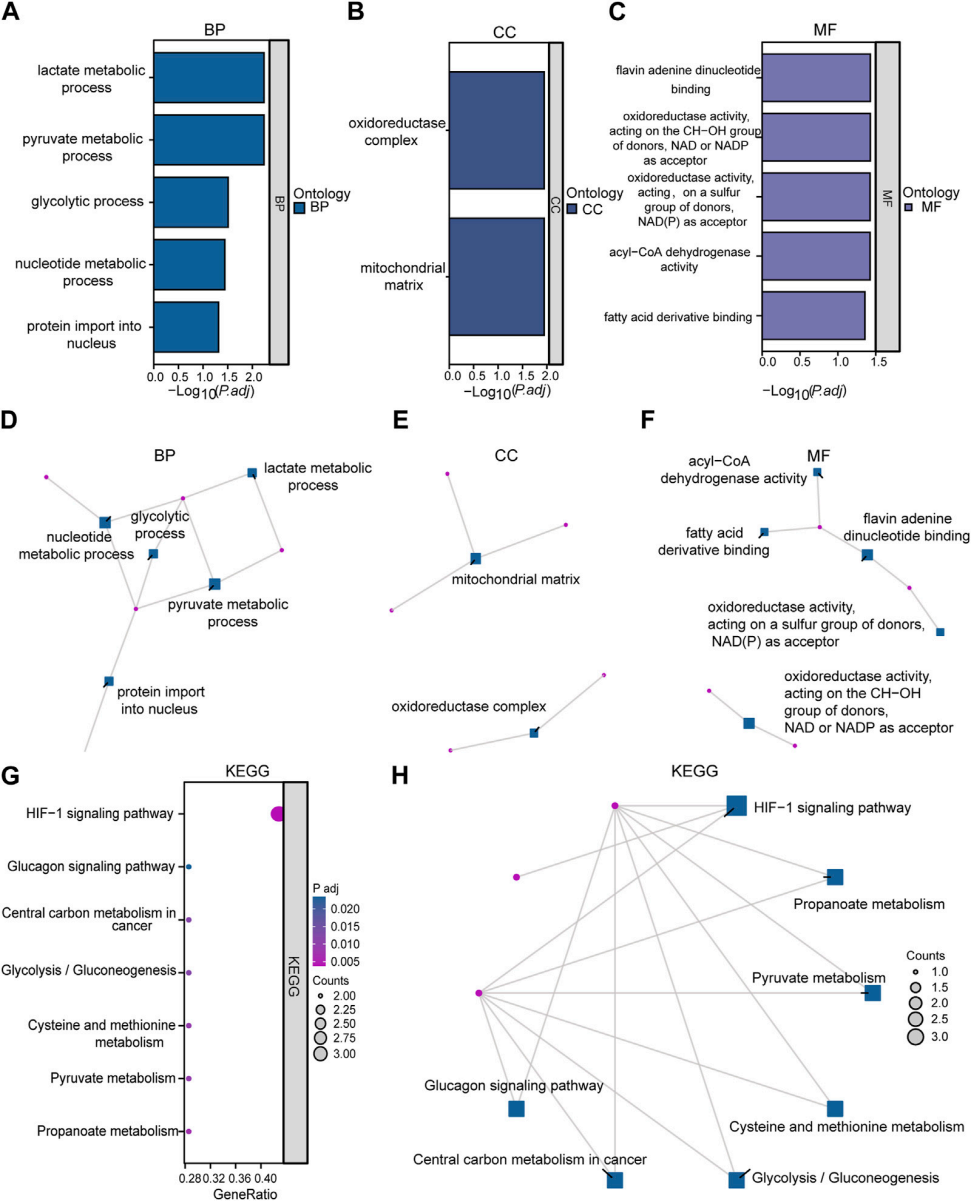
Enrichment analysis was performed on LMDEGs using GO and KEGG. **(A–C)** Column chart showing the results of GO functional enrichment analysis of LMDEGs, including BP **(A)**, CC **(B)**, and MF **(C)**. **(D–F)** Network diagrams displaying the results of GO functional enrichment analysis of LMDEGs in terms of BP **(D)**, CC **(E)**, and MF **(F)**. G-H. Bubble chart **(G)** and circular network diagram **(H)** showing the results of KEGG pathway enrichment analysis of LMDEGs. In the bubble chart **(G)**, the *y*-axis represents GO terms, bubble color represents adjusted *p*-values, and bubble size represents the number of molecules included in the GO terms. In the network diagrams **(D–F,H)** red dots represent specific genes, and blue squares represent specific pathways. The filtering criteria for GO/KEGG enrichment items were *P.adj* < 0.05 and *FDR* value (*q*-value) < 0.05. GO, Gene Ontology; BP, biological process; CC, cellular component; MF, molecular function; KEGG, Kyoto Encyclopedia of Genes and Genomes; LMDEGs, lactate metabolism-related differentially expressed genes.

Functional enrichment analysis by GO was performed on 10 LMDEGs (Table 3) to understand their biological significance. GO analysis revealed that the LMDEGs were enriched in various biological process (BP), molecular function (MF), and cellular component (CC) during septic shock. The BP analysis showed that the LMDEGs were enriched in metabolic processes, i.e., pyruvate, lactate, nucleotide, and glycolytic metabolism, as well as protein import into the nucleus. The MF enrichment analysis included oxidoreductase activity, acting on the CH-OH group of donors, NAD or NADP as acceptor, flavin adenine dinucleotide binding, acyl-CoA dehydrogenase activity, oxidoreductase activity, acting on a sulfur group of donors, NAD(P) as acceptor, and fatty acid derivative binding. The CC enrichment analysis showed that the oxidoreductase complex and the mitochondrial matrix were the LMDEGs’ principal sites of association. The results of the GO functional enrichment analysis were represented in Figures 4A–C, where the *y*-axis indicates GO terms, and the *x*-axis represents -log(P.adj). We also depicted the results of GO functional enrichment analysis in a network diagram (Figures 4D–F).

**TABLE 3:** GO enrichment analysis results.

Ontology	ID	Description	GeneRatio	BgRatio	pvalue	p.adjust	qvalue
BP	GO:0006090	pyruvate metabolic process	3/10	106/18800	2.0314E-05	0.005798302	0.003059748
BP	GO:0006089	lactate metabolic process	2/10	16/18800	3.04373E-05	0.005798302	0.003059748
BP	GO:0006096	glycolytic process	2/10	81/18800	0.00080678	0.03095373	0.0163342
BP	GO:0009117	nucleotide metabolic process	3/10	487/18800	0.0018098	0.03612573	0.01906345
BP	GO:0006606	protein import into nucleus	2/10	159/18800	0.003059286	0.04856616	0.025628227
CC	GO:0005759	mitochondrial matrix	3/10	473/19594	0.001478099	0.011346427	0.009384263
CC	GO:1990204	oxidoreductase complex	2/10	120/19594	0.001620918	0.011346427	0.009384263
MF	GO:0016616	oxidoreductase activity, acting on the CH-OH group of donors, NAD or NADP as acceptor	2/10	128/18410	0.002081045	0.037238383	0.016332624
MF	GO:0050660	flavin adenine dinucleotide binding	2/10	85/18410	0.000925507	0.037238383	0.016332624
MF	GO:0003995	acyl-CoA dehydrogenase activity	1/10	12/18410	0.006500695	0.037476529	0.016437074
MF	GO:0016668	oxidoreductase activity, acting on a sulfur group of donors, NAD(P) as acceptor	1/10	13/18410	0.007040699	0.037476529	0.016437074
MF	GO:1901567	fatty acid derivative binding	1/10	23/18410	0.012426228	0.043857276	0.019235647

A pathway enrichment analysis by KEGG database was performed on the 10 LMDEGs identified in the previous section (Table 4), and found that these genes were significantly enriched in 7 KEGG pathways, including the hypoxia-inducible factor-1 (HIF-1) signaling pathway, propanoate metabolism, pyruvate metabolism, and cysteine and methionine metabolism (Figure 4G). To better visualize the results, a circular network diagram was generated (Figure 4H).

**TABLE 4:** KEGG enrichment analysis results.

Ontology	ID	Description	GeneRatio	BgRatio	pvalue	p.adjust	qvalue
KEGG	hsa04066	HIF-1 signaling pathway	3/7	109/8164	7.79402E-05	0.003741129	0.002789438
KEGG	hsa00640	Propanoate metabolism	2/7	32/8164	0.000308783	0.007410786	0.005525586
KEGG	hsa00620	Pyruvate metabolism	2/7	47/8164	0.000668855	0.009451197	0.007046945
KEGG	hsa00270	Cysteine and methionine metabolism	2/7	51/8164	0.0007876	0.009451197	0.007046945
KEGG	hsa00010	Glycolysis/Gluconeogenesis	2/7	67/8164	0.001356873	0.01184197	0.008829539
KEGG	hsa05230	Central carbon metabolism in cancer	2/7	70/8164	0.001480246	0.01184197	0.008829539
KEGG	hsa04922	Glucagon signaling pathway	2/7	107/8164	0.003423654	0.023476483	0.017504395

### 3.3 GSEA

**FIGURE 5:**
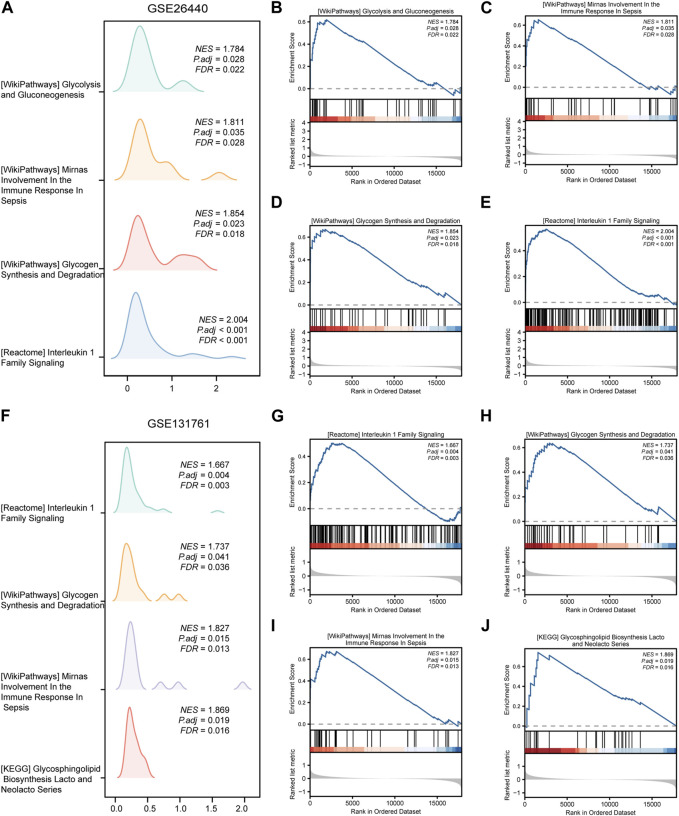
Results of gene set enrichment analysis (GSEA) on the septic shock datasets. **(A)** Four main biological features of GSEA in GSE26440. **(B–E)** Significant enrichment of DEGs in pathways such as glycolysis and gluconeogenesis **(B)**, miRNAs involvement in the immune response in sepsis **(C)**, glycogen synthesis and degradation **(D)**, reactome interleukin 1 family signaling **(E)** in the GSE26440 dataset. **(F)** Four main biological features of GSEA in GSE131761. **(G–J)** Significant enrichment of DEGs in pathways such as reactome interleukin 1 family signaling **(G)**, glycogen synthesis and degradation **(H)**, mirnas involvement in the immune response in sepsis **(I)**, glycosphingolipid biosynthesis lacto and neolacto series **(J)** in the GSE131761 dataset. The significant enrichment selection criterion for GSEA is *P.adj* < 0.05 and *FDR* value (*q*-value) < 0.05. GSEA, Gene Set Enrichment Analysis.

To assess the role of DEGs in the pathogenesis of septic shock, we conducted GSEA on two datasets, GSE26440 and GSE131761 and found that the DEGs in GSE26440 were significantly enriched in multiple pathways, including *WP GLYCOLYSIS AND GLUCONEOGENESIS, WP MIRNAS INVOLVEMENT IN THE IMMUNE RESPONSE IN SEPSIS, WP GLYCOGEN SYNTHESIS AND DEGRADATION,* and *REACTOME INTERLEUKIN 1 FAMILY SIGNALING*. The results were presented in Figures 5A–E and Table 5. Likewise, the DEGs in GSE131761 were significantly enriched in various pathways, i.e., *REACTOME INTERLEUKIN 1 FAMILY SIGNALING, WP GLYCOGEN SYNTHESIS AND DEGRADATION, WP MIRNAS INVOLVEMENT IN THE IMMUNE RESPONSE IN SEPSIS,* and *KEGG GLYCOSPHINGOLIPID BIOSYNTHESIS LACTO AND NEOLACTO SERIES*, as depicted in Figures 5F–J and Table 6.

**TABLE 5:** GSEA enrichment analysis results of GEO- GSE26440 dataset.

Description	setSize	NES	p.adjust	qvalues
REACTOME_INTERLEUKIN_1_FAMILY_SIGNALING	145	2.003516698	0.000115393	9.17738E-05
WP_GLYCOGEN_SYNTHESIS_AND_DEGRADATION	36	1.85401466	0.022659842	0.018021649
WP_NOTCH1_REGULATION_OF_ENDOTHELIAL_CELL_CALCIFICATION	17	1.814939099	0.029007368	0.023069914
WP_MIRNAS_INVOLVEMENT_IN_THE_IMMUNE_RESPONSE_IN_SEPSIS	36	1.811206797	0.034604798	0.027521618
WP_GLYCOLYSIS_AND_GLUCONEOGENESIS	44	1.783691927	0.027508443	0.021877801
REACTOME_NEUTROPHIL_DEGRANULATION	448	3.064982663	1.77071E-08	1.40827E-08
REACTOME_ACTIVATION_OF_MATRIX_METALLOPROTEINASES	31	2.370902154	2.86425E-08	2.27797E-08
REACTOME_COLLAGEN_DEGRADATION	64	2.257436488	2.17214E-05	1.72753E-05
WP_COMPLEMENT_SYSTEM	91	2.244919652	1.27922E-05	1.01738E-05
REACTOME_ANTIMICROBIAL_PEPTIDES	65	2.24184692	9.18658E-06	7.3062E-06
WP_MATRIX_METALLOPROTEINASES	29	2.229396828	5.14861E-05	4.09475E-05
WP_BURN_WOUND_HEALING	103	2.21615658	5.58363E-06	4.44073E-06
REACTOME_DEGRADATION_OF_THE_EXTRACELLULAR_MATRIX	138	2.189833983	1.57087E-06	1.24933E-06
REACTOME_INTERLEUKIN_4_AND_INTERLEUKIN_13_SIGNALING	106	2.171320372	3.27691E-05	2.60616E-05
REACTOME_RHO_GTPASES_ACTIVATE_NADPH_OXIDASES	24	2.153439893	0.000443012	0.000352333
NABA_ECM_REGULATORS	226	2.11968437	2.3532E-07	1.87153E-07
REACTOME_EXTRACELLULAR_MATRIX_ORGANIZATION	290	2.112971131	1.77071E-08	1.40827E-08
WP_MICROGLIA_PATHOGEN_PHAGOCYTOSIS_PATHWAY	40	2.111001717	0.000317917	0.000252843
WP_LTF_DANGER_SIGNAL_RESPONSE_PATHWAY	19	2.107592082	0.000228875	0.000182027
REACTOME_RESPONSE_TO_ELEVATED_PLATELET_CYTOSOLIC_CA2	124	2.10371762	1.44602E-05	1.15004E-05

**TABLE 6:** GSEA enrichment analysis results of GEO- GSE131761 dataset.

Description	setSize	NES	p.adjust	qvalues
WP_ATM_SIGNALING_PATHWAY	37	1.910403	0.003677	0.003202
KEGG_GLYCOSPHINGOLIPID_BIOSYNTHESIS_LACTO_AND_NEOLACTO_SERIES	25	1.868707	0.018703	0.016285
WP_MIRNAS_INVOLVEMENT_IN_THE_IMMUNE_RESPONSE_IN_SEPSIS	37	1.827298	0.014556	0.012674
WP_GLYCOGEN_SYNTHESIS_AND_DEGRADATION	39	1.737055	0.040929	0.035638
REACTOME_INTERLEUKIN_1_FAMILY_SIGNALING	150	1.66698	0.00363	0.00316
REACTOME_NEUTROPHIL_DEGRANULATION	459	2.373541	1.47E-08	1.28E-08
REACTOME_CELL_CYCLE_CHECKPOINTS	242	1.93119	2.66E-07	2.32E-07
REACTOME_ANTIMICROBIAL_PEPTIDES	84	1.926725	0.000322	0.00028
REACTOME_RHO_GTPASE_EFFECTORS	253	1.906874	6.94E-07	6.04E-07
REACTOME_G1_S_SPECIFIC_TRANSCRIPTION	29	1.903141	0.003677	0.003202
REACTOME_CELL_CYCLE_MITOTIC	479	1.887184	1.47E-08	1.28E-08
KEGG_P53_SIGNALING_PATHWAY	65	1.862469	0.003433	0.002989
REACTOME_UNWINDING_OF_DNA	12	1.844032	0.011889	0.010352
PID_RHOA_PATHWAY	43	1.832345	0.008903	0.007752
REACTOME_G2_M_CHECKPOINTS	127	1.830426	0.000329	0.000287
REACTOME_RESOLUTION_OF_SISTER_CHROMATID_COHESION	115	1.823666	0.000435	0.000379
REACTOME_MITOTIC_PROMETAPHASE	190	1.823268	4.08E-05	3.55E-05
REACTOME_ER_TO_GOLGI_ANTEROGRADE_TRANSPORT	149	1.819755	0.00023	0.000201
BIOCARTA_MPR_PATHWAY	21	1.819122	0.015684	0.013656
BIOCARTA_UCALPAIN_PATHWAY	14	1.817549	0.014556	0.012674

### 3.4 Protein-protein, mRNA-miRNA, mRNA-RBP, and mRNA-TF interaction networks of LMDEGs

**FIGURE 6:**
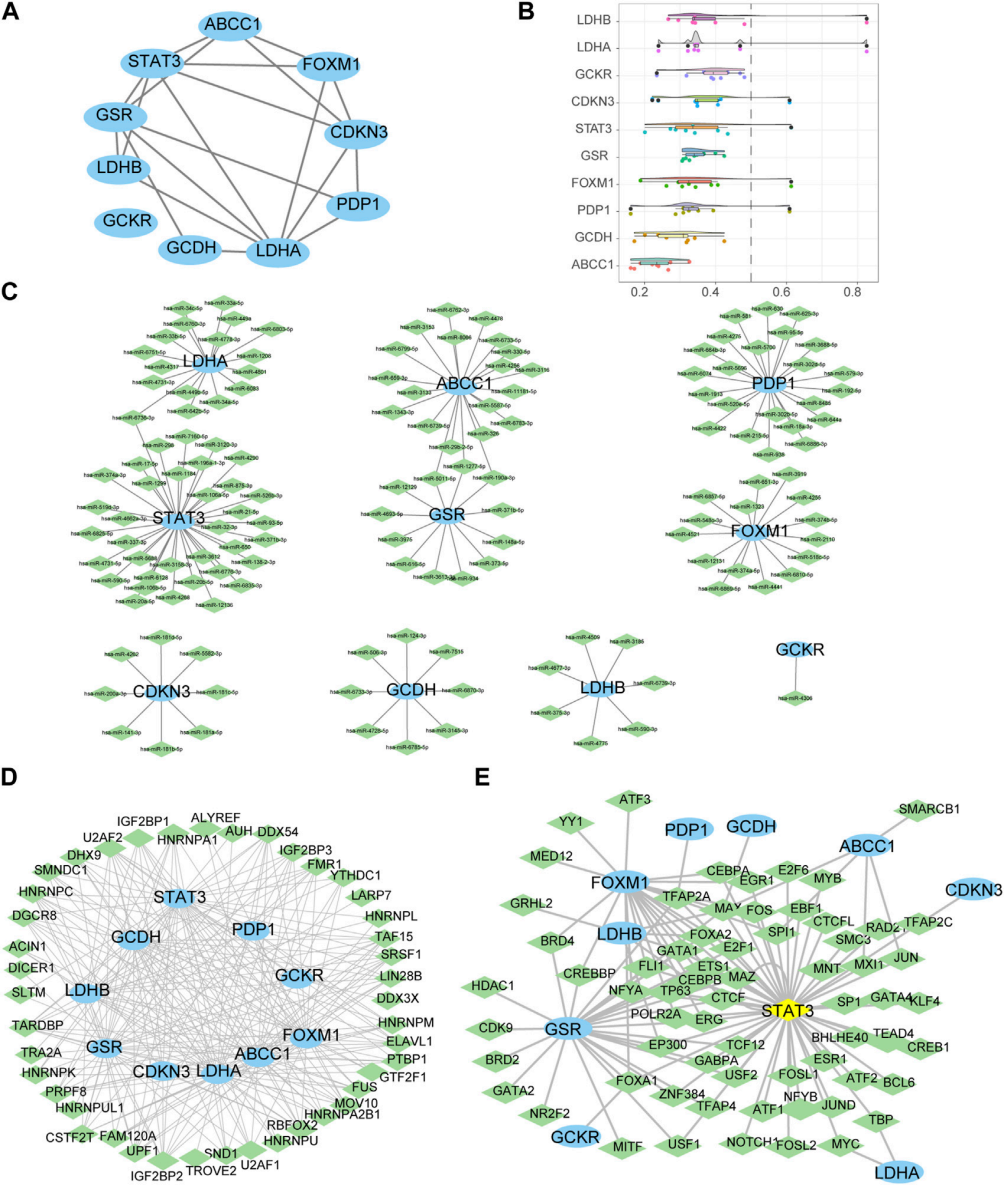
Construction of protein-protein, mRNA-miRNA, mRNA-RBP, and mRNA-TF interaction networks for the LMDEGs, as well as their correlation analysis. **(A)** PPI network of LMDEGs. **(B)** Friends analysis results of LMDEGs. C-E. mRNA-miRNA **(C)**, mRNA-RBP **(D)**, and mRNA-TF **(E)** interaction networks of LMDEGs. In the mRNA-miRNA interaction network **(C)**, mRNA is represented by light blue ellipse and miRNA by light green diamond. In the mRNA-RBP interaction network **(D)**, mRNA is represented by light blue circle and RBP by light green diamond. In the mRNA-TF interaction network **(E)**, mRNA is represented by light blue circle, transcription factors (TFs) by light green diamond, and yellow diamond represents mRNA that is also a TF. PPI, Protein-protein interaction; RBP, RNA binding protein; TF, Transcription factors.

PPI analysis was conducted on 10 LMDEGs obtained from the GSE26440 and GSE131761 datasets via the STRING database. Cytoscape was used to construct PPI networks for these genes, providing a visual representation of their interaction relationships (Figure 6A). Additionally, To determine the semantic similarity of GO terms, sets of GO terms, gene products, and gene clusters among the LMDEGs, we used the R package GOSemSim, and a boxplot was then used to show the commonalities that resulted from this (Figure 6B).

In order to investigate the regulatory mechanisms of LMDEGs, we utilized various bioinformatic tools and databases. Firstly, we employed the miRDB database to predict potential miRNAs that could interact with the LMDEGs. Subsequently, we visualized the mRNA-miRNA interaction network using Cytoscape software, which consisted of 10 LMDEGs and 144 miRNA molecules, resulting in 148 pairs of mRNA-miRNA interaction relationships (Figure 6C). The specific details of these interactions are provided in Table 7.

**TABLE 7:** mRNA-miRNA interaction network nodes.

mRNA		miRNA	mRNA		miRNA
LDHB	-	hsa-miR-590-3p	FOXM1	-	hsa-miR-1323
LDHB	-	hsa-miR-4775	FOXM1	-	hsa-miR-4255
LDHB	-	hsa-miR-375-3p	FOXM1	-	hsa-miR-6810-5p
LDHB	-	hsa-miR-6739-3p	FOXM1	-	hsa-miR-3919
LDHB	-	hsa-miR-4509	FOXM1	-	hsa-miR-518c-5p
LDHB	-	hsa-miR-4677-3p	FOXM1	-	hsa-miR-548o-3p
LDHB	-	hsa-miR-3185	FOXM1	-	hsa-miR-651-3p
STAT3	-	hsa-miR-1299	FOXM1	-	hsa-miR-6869-5p
STAT3	-	hsa-miR-374a-3p	FOXM1	-	hsa-miR-374a-5p
STAT3	-	hsa-miR-7160-5p	FOXM1	-	hsa-miR-6857-5p
STAT3	-	hsa-miR-21-5p	FOXM1	-	hsa-miR-4441
STAT3	-	hsa-miR-590-5p	FOXM1	-	hsa-miR-374b-5p
STAT3	-	hsa-miR-6825-5p	FOXM1	-	hsa-miR-2110
STAT3	-	hsa-miR-4268	PDP1	-	hsa-miR-18a-3p
STAT3	-	hsa-miR-196a-1-3p	PDP1	-	hsa-miR-938
STAT3	-	hsa-miR-32-3p	PDP1	-	hsa-miR-6886-3p
STAT3	-	hsa-miR-6736-3p	PDP1	-	hsa-miR-5696
STAT3	-	hsa-miR-6128	PDP1	-	hsa-miR-4275
STAT3	-	hsa-miR-337-3p	PDP1	-	hsa-miR-1913
STAT3	-	hsa-miR-875-3p	PDP1	-	hsa-miR-579-3p
STAT3	-	hsa-miR-6835-3p	PDP1	-	hsa-miR-664b-3p
STAT3	-	hsa-miR-371b-3p	PDP1	-	hsa-miR-520e-5p
STAT3	-	hsa-miR-106b-5p	PDP1	-	hsa-miR-644a
STAT3	-	hsa-miR-526b-3p	PDP1	-	hsa-miR-95-5p
STAT3	-	hsa-miR-20b-5p	PDP1	-	hsa-miR-5700
STAT3	-	hsa-miR-106a-5p	PDP1	-	hsa-miR-625-3p
STAT3	-	hsa-miR-93-5p	PDP1	-	hsa-miR-4422
STAT3	-	hsa-miR-17-5p	PDP1	-	hsa-miR-630
STAT3	-	hsa-miR-20a-5p	PDP1	-	hsa-miR-302b-5p
STAT3	-	hsa-miR-519d-3p	PDP1	-	hsa-miR-302d-5p
STAT3	-	hsa-miR-3120-3p	PDP1	-	hsa-miR-6074
STAT3	-	hsa-miR-1184	PDP1	-	hsa-miR-581
STAT3	-	hsa-miR-3612	PDP1	-	hsa-miR-3688-5p
STAT3	-	hsa-miR-650	PDP1	-	hsa-miR-192-5p
STAT3	-	hsa-miR-4662a-3p	PDP1	-	hsa-miR-215-5p
STAT3	-	hsa-miR-138-2-3p	PDP1	-	hsa-miR-8485
STAT3	-	hsa-miR-4290	GCDH	-	hsa-miR-6870-3p
STAT3	-	hsa-miR-4731-5p	GCDH	-	hsa-miR-124-3p
STAT3	-	hsa-miR-5688	GCDH	-	hsa-miR-506-3p
STAT3	-	hsa-miR-6776-3p	GCDH	-	hsa-miR-3145-3p
STAT3	-	hsa-miR-3158-3p	GCDH	-	hsa-miR-6785-5p
STAT3	-	hsa-miR-298	GCDH	-	hsa-miR-7515
STAT3	-	hsa-miR-12136	GCDH	-	hsa-miR-4728-5p
LDHA	-	hsa-miR-1208	GCDH	-	hsa-miR-6733-3p
LDHA	-	hsa-miR-4801	GCKR	-	hsa-miR-4306
LDHA	-	hsa-miR-6751-5p	ABCC1	-	hsa-miR-5011-5p
LDHA	-	hsa-miR-4731-3p	ABCC1	-	hsa-miR-6783-3p
LDHA	-	hsa-miR-4778-3p	ABCC1	-	hsa-miR-1343-3p
LDHA	-	hsa-miR-642b-5p	ABCC1	-	hsa-miR-659-3p
LDHA	-	hsa-miR-449a	ABCC1	-	hsa-miR-29b-2-5p
LDHA	-	hsa-miR-34a-5p	ABCC1	-	hsa-miR-3133
LDHA	-	hsa-miR-33b-5p	ABCC1	-	hsa-miR-5587-5p
LDHA	-	hsa-miR-6760-3p	ABCC1	-	hsa-miR-6762-3p
LDHA	-	hsa-miR-33a-5p	ABCC1	-	hsa-miR-330-5p
LDHA	-	hsa-miR-6803-5p	ABCC1	-	hsa-miR-6799-5p
LDHA	-	hsa-miR-6083	ABCC1	-	hsa-miR-326
LDHA	-	hsa-miR-34c-5p	ABCC1	-	hsa-miR-4478
LDHA	-	hsa-miR-449b-5p	ABCC1	-	hsa-miR-6733-5p
LDHA	-	hsa-miR-6736-3p	ABCC1	-	hsa-miR-3153
LDHA	-	hsa-miR-4317	ABCC1	-	hsa-miR-6739-5p
GSR	-	hsa-miR-5011-5p	ABCC1	-	hsa-miR-8066
GSR	-	hsa-miR-190a-3p	ABCC1	-	hsa-miR-190a-3p
GSR	-	hsa-miR-148a-5p	ABCC1	-	hsa-miR-3116
GSR	-	hsa-miR-1277-5p	ABCC1	-	hsa-miR-4256
GSR	-	hsa-miR-3613-3p	ABCC1	-	hsa-miR-1277-5p
GSR	-	hsa-miR-934	ABCC1	-	hsa-miR-11181-5p
GSR	-	hsa-miR-4693-5p	CDKN3	-	hsa-miR-181c-5p
GSR	-	hsa-miR-371b-5p	CDKN3	-	hsa-miR-181d-5p
GSR	-	hsa-miR-616-5p	CDKN3	-	hsa-miR-4262
GSR	-	hsa-miR-373-5p	CDKN3	-	hsa-miR-181a-5p
GSR	-	hsa-miR-3975	CDKN3	-	hsa-miR-181b-5p
GSR	-	hsa-miR-12129	CDKN3	-	hsa-miR-5582-3p
FOXM1	-	hsa-miR-4521	CDKN3	-	hsa-miR-141-3p
FOXM1	-	hsa-miR-12131	CDKN3	-	hsa-miR-200a-3p

Next, we utilized the ENCORI database to predict RBPs that may interact with the LMDEGs, and we constructed the mRNA-RBP interaction network by Cytoscape software. This network was comprised of 10 LMDEGs and 43 RBP molecules, which formed a total of 243 pairs of mRNA-RBP interaction relationships (Figure 6D). The detailed information regarding these interactions is listed in Table 8.

**TABLE 8:** mRNA-RBP interaction network nodes.

mRNA		RBP	mRNA		RBP	mRNA		RBP
ABCC1	-	ACIN1	GCDH	-	HNRNPK	LDHA	-	TARDBP
ABCC1	-	ALYREF	GCDH	-	HNRNPM	LDHA	-	TRA2A
ABCC1	-	CSTF2T	GCDH	-	HNRNPU	LDHA	-	TROVE2
ABCC1	-	DDX54	GCDH	-	IGF2BP2	LDHA	-	U2AF1
ABCC1	-	DGCR8	GCDH	-	LIN28B	LDHA	-	U2AF2
ABCC1	-	DHX9	GCDH	-	MOV10	LDHA	-	YTHDC1
ABCC1	-	ELAVL1	GCDH	-	PRPF8	LDHB	-	ACIN1
ABCC1	-	FAM120A	GCDH	-	RBFOX2	LDHB	-	CSTF2T
ABCC1	-	FMR1	GCDH	-	SMNDC1	LDHB	-	DGCR8
ABCC1	-	FUS	GCDH	-	SND1	LDHB	-	ELAVL1
ABCC1	-	HNRNPA2B1	GCDH	-	SRSF1	LDHB	-	FMR1
ABCC1	-	HNRNPC	GCDH	-	TAF15	LDHB	-	FUS
ABCC1	-	HNRNPL	GCDH	-	TARDBP	LDHB	-	HNRNPA1
ABCC1	-	HNRNPM	GCDH	-	U2AF1	LDHB	-	HNRNPA2B1
ABCC1	-	IGF2BP1	GCDH	-	U2AF2	LDHB	-	HNRNPC
ABCC1	-	IGF2BP2	GCDH	-	UPF1	LDHB	-	HNRNPL
ABCC1	-	IGF2BP3	GCKR	-	PRPF8	LDHB	-	IGF2BP1
ABCC1	-	LIN28B	GCKR	-	TAF15	LDHB	-	IGF2BP2
ABCC1	-	MOV10	GCKR	-	U2AF2	LDHB	-	IGF2BP3
ABCC1	-	PRPF8	GSR	-	CSTF2T	LDHB	-	LIN28B
ABCC1	-	PTBP1	GSR	-	DDX54	LDHB	-	MOV10
ABCC1	-	RBFOX2	GSR	-	DGCR8	LDHB	-	PTBP1
ABCC1	-	SND1	GSR	-	DHX9	LDHB	-	RBFOX2
ABCC1	-	SRSF1	GSR	-	DICER1	LDHB	-	SRSF1
ABCC1	-	TAF15	GSR	-	ELAVL1	LDHB	-	TAF15
ABCC1	-	TARDBP	GSR	-	FAM120A	LDHB	-	TARDBP
ABCC1	-	U2AF1	GSR	-	FMR1	LDHB	-	U2AF2
ABCC1	-	U2AF2	GSR	-	FUS	LDHB	-	UPF1
ABCC1	-	UPF1	GSR	-	HNRNPA1	LDHB	-	YTHDC1
ABCC1	-	YTHDC1	GSR	-	HNRNPC	PDP1	-	CSTF2T
CDKN3	-	DDX54	GSR	-	HNRNPK	PDP1	-	DDX54
CDKN3	-	HNRNPA1	GSR	-	HNRNPM	PDP1	-	ELAVL1
CDKN3	-	HNRNPC	GSR	-	IGF2BP1	PDP1	-	FMR1
CDKN3	-	HNRNPM	GSR	-	IGF2BP2	PDP1	-	HNRNPA2B1
CDKN3	-	IGF2BP1	GSR	-	IGF2BP3	PDP1	-	HNRNPC
CDKN3	-	IGF2BP2	GSR	-	LARP7	PDP1	-	HNRNPM
CDKN3	-	PRPF8	GSR	-	LIN28B	PDP1	-	IGF2BP2
CDKN3	-	RBFOX2	GSR	-	MOV10	PDP1	-	IGF2BP3
CDKN3	-	SRSF1	GSR	-	PRPF8	PDP1	-	MOV10
CDKN3	-	TARDBP	GSR	-	PTBP1	PDP1	-	RBFOX2
CDKN3	-	U2AF2	GSR	-	RBFOX2	PDP1	-	TAF15
CDKN3	-	UPF1	GSR	-	SMNDC1	PDP1	-	TARDBP
FOXM1	-	ALYREF	GSR	-	SND1	PDP1	-	U2AF2
FOXM1	-	CSTF2T	GSR	-	SRSF1	PDP1	-	UPF1
FOXM1	-	DDX54	GSR	-	TAF15	STAT3	-	AUH
FOXM1	-	DGCR8	GSR	-	TARDBP	STAT3	-	CSTF2T
FOXM1	-	DHX9	GSR	-	TRA2A	STAT3	-	DDX54
FOXM1	-	ELAVL1	GSR	-	U2AF1	STAT3	-	DGCR8
FOXM1	-	FAM120A	GSR	-	U2AF2	STAT3	-	DHX9
FOXM1	-	FMR1	GSR	-	UPF1	STAT3	-	ELAVL1
FOXM1	-	FUS	LDHA	-	ACIN1	STAT3	-	FAM120A
FOXM1	-	GTF2F1	LDHA	-	AUH	STAT3	-	FMR1
FOXM1	-	HNRNPA1	LDHA	-	CSTF2T	STAT3	-	FUS
FOXM1	-	HNRNPC	LDHA	-	DDX54	STAT3	-	GTF2F1
FOXM1	-	HNRNPK	LDHA	-	DGCR8	STAT3	-	HNRNPA1
FOXM1	-	IGF2BP1	LDHA	-	DHX9	STAT3	-	HNRNPA2B1
FOXM1	-	IGF2BP2	LDHA	-	ELAVL1	STAT3	-	HNRNPC
FOXM1	-	IGF2BP3	LDHA	-	FAM120A	STAT3	-	HNRNPK
FOXM1	-	LIN28B	LDHA	-	FMR1	STAT3	-	HNRNPL
FOXM1	-	MOV10	LDHA	-	FUS	STAT3	-	HNRNPM
FOXM1	-	PRPF8	LDHA	-	GTF2F1	STAT3	-	HNRNPU
FOXM1	-	RBFOX2	LDHA	-	HNRNPA1	STAT3	-	HNRNPUL1
FOXM1	-	SMNDC1	LDHA	-	HNRNPA2B1	STAT3	-	IGF2BP1
FOXM1	-	SRSF1	LDHA	-	HNRNPC	STAT3	-	IGF2BP2
FOXM1	-	TAF15	LDHA	-	HNRNPK	STAT3	-	IGF2BP3
FOXM1	-	TARDBP	LDHA	-	HNRNPM	STAT3	-	LIN28B
FOXM1	-	U2AF1	LDHA	-	HNRNPU	STAT3	-	MOV10
FOXM1	-	U2AF2	LDHA	-	HNRNPUL1	STAT3	-	PRPF8
FOXM1	-	UPF1	LDHA	-	IGF2BP1	STAT3	-	PTBP1
FOXM1	-	YTHDC1	LDHA	-	IGF2BP2	STAT3	-	RBFOX2
GCDH	-	CSTF2T	LDHA	-	IGF2BP3	STAT3	-	SLTM
GCDH	-	DDX3X	LDHA	-	LIN28B	STAT3	-	SMNDC1
GCDH	-	DDX54	LDHA	-	MOV10	STAT3	-	SND1
GCDH	-	DGCR8	LDHA	-	PRPF8	STAT3	-	SRSF1
GCDH	-	DHX9	LDHA	-	PTBP1	STAT3	-	TAF15
GCDH	-	ELAVL1	LDHA	-	RBFOX2	STAT3	-	TARDBP
GCDH	-	FMR1	LDHA	-	SLTM	STAT3	-	TRA2A
GCDH	-	GTF2F1	LDHA	-	SMNDC1	STAT3	-	U2AF1
GCDH	-	HNRNPA1	LDHA	-	SND1	STAT3	-	U2AF2
GCDH	-	HNRNPA2B1	LDHA	-	SRSF1	STAT3	-	UPF1
GCDH	-	HNRNPC	LDHA	-	TAF15	STAT3	-	YTHDC1

Additionally, we searched the CHIPBase and hTFtarget databases to identify TFs that could potentially interact with the LMDEGs. A total of 67 TFs were identified to have interaction relationships with the LMDEGs, and the mRNA-TF interaction network was constructed by Cytoscape software, consisting of 10 LMDEGs and 67 TFs. In total, 441 pairs of mRNA-TF interaction relationships were observed, with STAT3 showing the most interactions with TFs, comprising 54 pairs of mRNA-TF interaction relationships, since STAT3 was both a gene and a TF (Figure 6E). The detailed mRNA-TF interaction relationships are presented in Table 9.

**TABLE 9:** mRNA-TF interaction network nodes.

mRNA		TF	mRNA		TF
ABCC1	-	JUN	LDHB	-	FOXA2
ABCC1	-	MAX	LDHB	-	GATA1
ABCC1	-	MXI1	LDHB	-	GRHL2
ABCC1	-	SMARCB1	LDHB	-	MAX
ABCC1	-	SPI1	PDP1	-	TFAP2A
CDKN3	-	TFAP2C	STAT3	-	CREB1
FOXM1	-	ATF3	STAT3	-	CTCF
FOXM1	-	BRD4	STAT3	-	CTCFL
FOXM1	-	CEBPA	STAT3	-	E2F1
FOXM1	-	CEBPB	STAT3	-	E2F6
FOXM1	-	CREBBP	STAT3	-	EBF1
FOXM1	-	CTCF	STAT3	-	EGR1
FOXM1	-	E2F1	STAT3	-	EP300
FOXM1	-	EGR1	STAT3	-	ERG
FOXM1	-	ERG	STAT3	-	ESR1
FOXM1	-	ETS1	STAT3	-	ETS1
FOXM1	-	FLI1	STAT3	-	FLI1
FOXM1	-	FOS	STAT3	-	FOS
FOXM1	-	FOXA1	STAT3	-	FOSL1
FOXM1	-	FOXA2	STAT3	-	FOSL2
FOXM1	-	MAX	STAT3	-	FOXA1
FOXM1	-	MAZ	STAT3	-	FOXA2
FOXM1	-	MED12	STAT3	-	GABPA
FOXM1	-	NFYA	STAT3	-	GATA1
FOXM1	-	POLR2A	STAT3	-	GATA4
FOXM1	-	SPI1	STAT3	-	ATF1
FOXM1	-	YY1	STAT3	-	ATF2
GCDH	-	MAX	STAT3	-	JUN
GCKR	-	FOXA1	STAT3	-	JUND
GSR	-	CTCF	STAT3	-	KLF4
GSR	-	E2F1	STAT3	-	MAX
GSR	-	EP300	STAT3	-	MAZ
GSR	-	ERG	STAT3	-	BCL6
GSR	-	ETS1	STAT3	-	MNT
GSR	-	FLI1	STAT3	-	MXI1
GSR	-	FOXA1	STAT3	-	MYB
GSR	-	FOXA2	STAT3	-	MYC
GSR	-	GABPA	STAT3	-	BHLHE40
GSR	-	GATA1	STAT3	-	NFYA
GSR	-	GATA2	STAT3	-	NFYB
GSR	-	HDAC1	STAT3	-	NOTCH1
GSR	-	MAX	STAT3	-	POLR2A
GSR	-	MAZ	STAT3	-	RAD21
GSR	-	MITF	STAT3	-	SMC3
GSR	-	NR2F2	STAT3	-	SP1
GSR	-	BRD2	STAT3	-	SPI1
GSR	-	BRD4	STAT3	-	STAT3
GSR	-	TCF12	STAT3	-	TBP
GSR	-	TFAP2A	STAT3	-	TCF12
GSR	-	TFAP4	STAT3	-	TEAD4
GSR	-	CDK9	STAT3	-	TFAP2A
GSR	-	USF1	STAT3	-	TFAP2C
GSR	-	USF2	STAT3	-	TFAP4
GSR	-	CEBPB	STAT3	-	TP63
GSR	-	ZNF384	STAT3	-	USF1
GSR	-	CREBBP	STAT3	-	USF2
LDHA	-	MYC	STAT3	-	CEBPA
LDHA	-	TBP	STAT3	-	CEBPB
LDHB	-	CEBPB	STAT3	-	ZNF384
LDHB	-	EP300			

### 3.5 Expression differences and diagnostic performance of LMDEGs

**FIGURE 7:**
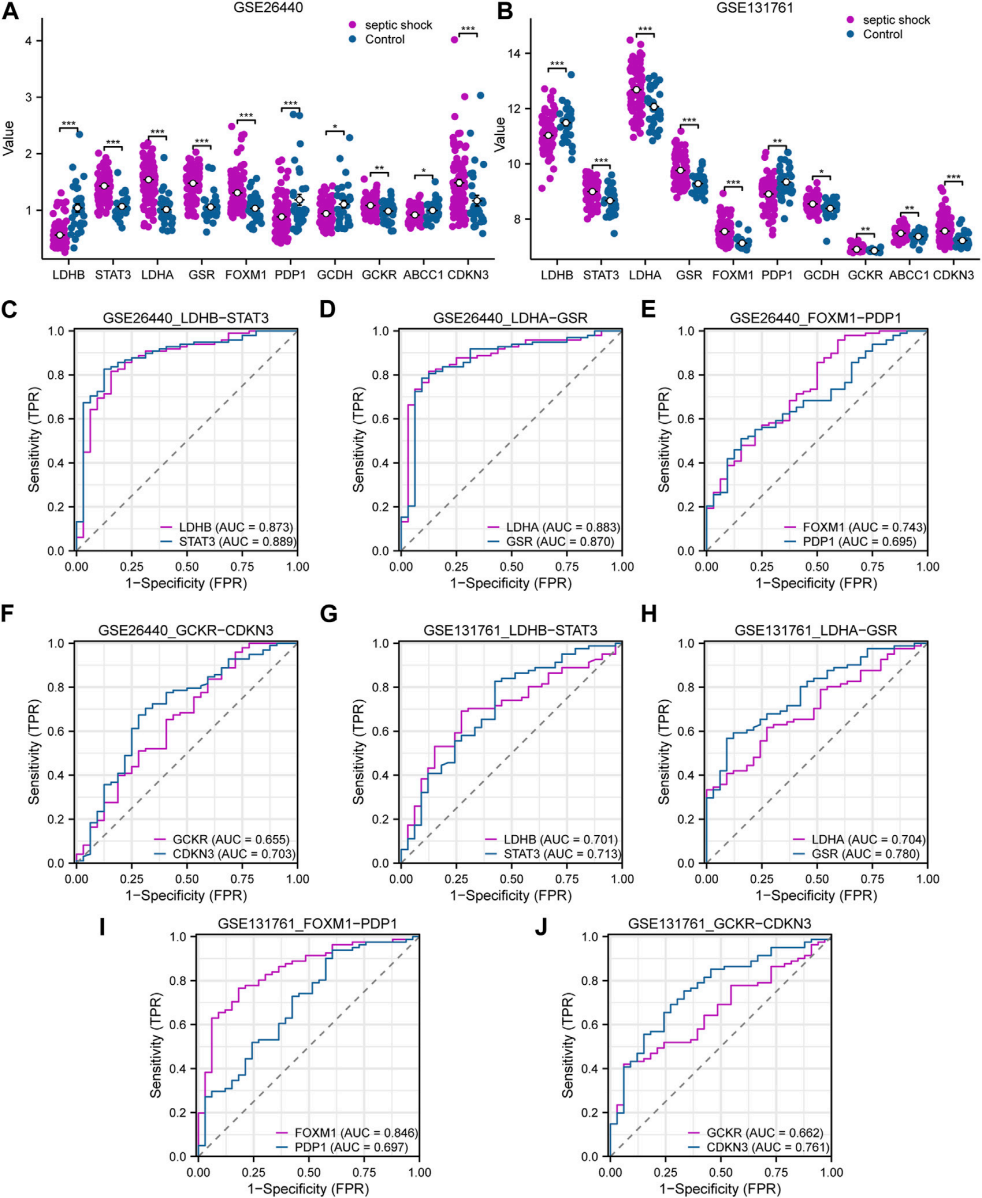
Expression differences and diagnostic performance of LMDEGs in the GSE26440 and GSE131761 datasets. **(A)** Differential expression analysis of LMDEGs in the GSE26440 dataset. **(B)** Differential expression analysis of LMDEGs in the GSE131761 dataset. C-F. ROC curve results for LMDEGs: *LDHB, STAT3* **(C)**, *LDHA*, *GSR* **(D)**, *FOXM1*, *PDP1* **(E)**, and *GCKR*, *CDKN3* **(F)** in the GSE26440 dataset. G-J. ROC curve results for LMDEGs: *LDHB, STAT3* **(G)**, *LDHA*, *GSR* **(H)**, *FOXM1*, *PDP1* **(I)**, and *GCKR*, *CDKN3* **(J)** in the GSE131761 dataset. ns indicates no statistical significance; *p < 0.05; **p < 0.01; ***p < 0.001. An AUC (range from 0.5 to 1) closer to 1 in the ROC curve indicates better diagnostic performance. ROC, receiver operating characteristic curve; AUC, Area Under Curve.

In this study, we conducted differential expression analysis on 10 LMDEGs in two datasets (GSE26440 and GSE131761) to identify significant expression differences between septic shock patients and normal controls or non-septic shock patients. Specifically, we performed the Wilcoxon signed rank test to evaluate the expression levels of the 10 LMDEGs in septic shock group and control group in the GSE26440 dataset. Our analysis found significant expression differences (*p* < 0.001 for LDHB, STAT3, LDHA, GSR, FOXM1, and CDKN3; *p* < 0.01 for GCKR; Figure 7A) among all 10 LMDEGs between the two groups. We also examined the expression levels of the 10 LMDEGs in the GSE131761 dataset, comparing septic shock group and non-septic shock patients group, and identified significant expression differences (*p* < 0.001 for *LDHB, STAT3, LDHA, GSR, FOXM1,* and *CDKN3*; *p* < 0.01 for *PDP1, GCDH, GCKR,* and *ABCC1*) between the two groups for all 10 LMDEGs (Figure 7B).

Based on the comparison of group distributions, we generated ROC curves for eight DEGs associated with lactate metabolism exhibiting similar expression trends in both the GSE26440 and GSE131761 datasets. The ROC curves were presented in Figures 7C–F. Notably, the following genes were observed to show a certain correlation with the occurrence of septic shock in the GSE26440 dataset: *LDHB* (AUC = 0.873, red line in Figure 7C), *STAT3* (AUC = 0.889, blue line in Figure 7C), *LDHA* (AUC = 0.883, red line in Figure 7D), *GSR* (AUC = 0.870, blue line in Figure 7D), *FOXM1* (AUC = 0.743, red line in Figure 7E), and *CDKN3* (AUC = 0.703, blue line in Figure 7F). Conversely, the expression of *PDP1* (AUC = 0.695, blue line in Figure 7E) and *GCKR* (AUC = 0.655, red line in Figure 7F) genes in the GSE26440 dataset exhibited a low correlation with septic shock occurrence. ROC curve analysis in the GSE131761 dataset revealed a similar correlation pattern. Specifically, the following genes exhibited a certain correlation with septic shock occurrence: *LDHB* (AUC = 0.701, red line in Figure 7G), *STAT3* (AUC = 0.713, blue line in Figure 7G), *LDHA* (AUC = 0.704, red line in Figure 7H), *GSR* (AUC = 0.780, blue line in Figure 7H), *FOXM1* (AUC = 0.846, red line in Figure 7I), and *CDKN3* (AUC = 0.761, blue line in Figure 7J). Meanwhile, *PDP1* (AUC = 0.697, blue line in Figure 7I) and *GCKR* (AUC = 0.662, red line in Figure 7J) genes in the GSE131761 dataset exhibited a low correlation with septic shock occurrence.

### 3.6 Immune characteristic analysis by ssGSEA

**FIGURE 8:**
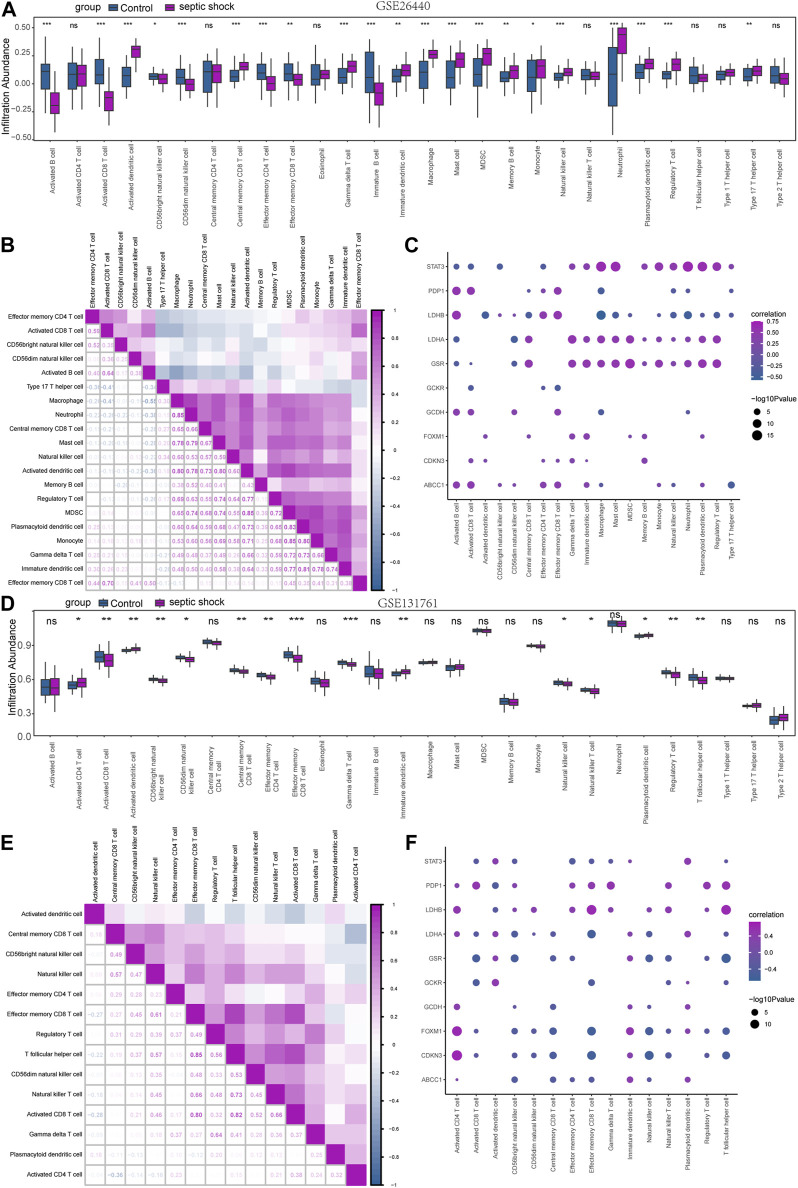
Immune infiltration analysis of the GSE26440 and GSE131761 datasets by ssGSEA. **(A)** ssGSEA immune infiltration analysis results in GSE26440. **(B)** Correlation analysis results of immune cell infiltration abundance in GSE26440. **(C)** Correlation heatmap of immune cells and LMDEGs in GSE26440 dataset. **(D)** ssGSEA immune infiltration analysis results for DEGs in GSE131761. **(E)** Correlation analysis results of immune cell infiltration abundance in GSE131761. **(F)** Correlation heatmap of immune cells and LMDEGs in GSE131761 dataset. ns indicates no statistical significance. *p < 0.05; **p < 0.01; ***p < 0.001. ssGSEA: single-sample gene-set enrichment analysis.

In order to explore the immunological characteristics of LMDEGs in septic shock, we employed ssGSEA to estimate the infiltration abundance of 28 distinct immune cells in septic shock and control groups. The differential gene expression analysis in the GSE26440 dataset was used for this purpose. We compared the infiltration differences of the 28 immune cells between septic shock and control groups using the Mann-Whitney *U* test and illustrated the results in a grouping comparison chart (Figure 8A). Our findings indicated that 21 immune cell types demonstrated statistically significant differences in infiltration abundance between septic shock and control groups (*p* < 0.05). Additionally, we analyzed the correlations between the infiltration abundance of these 21 immune cells and presented the results in Figure 8B, which showed generally positive correlations between the infiltration abundance of these immune cells.

Furthermore, we examined the relationship between the infiltration abundance of the previously mentioned 21 immune cells and the expression of 10 LMDEGs in GSE26440 dataset. We applied a significance threshold of *p* < 0.05 for the correlation analysis results (Figure 8C). Our findings indicated noteworthy associations between the infiltration abundance of 20 immune cells and the 10 LMDEGs (*p* < 0.05), with most of these associations being positively correlated. Importantly, we observed that *STAT3, LDHB, LDHA* and *GSR* displayed the most substantial correlations with the infiltration abundance of the 20 immune cells.

Similarly, we employed the ssGSEA algorithm to determine the infiltration abundance of 28 immune cells in septic shock and non-septic shock group samples obtained from the GSE131761 dataset. We performed a Mann-Whitney *U* test to analyze the infiltration differences of the 28 immune cells between the two groups and presented the results through a grouping comparison chart (Figure 8D). Our analysis revealed statistically significant differences in the infiltration abundance of 15 immune cell types (*p* < 0.05) between the septic shock group and non-septic shock patients group.

We subsequently conducted correlation analyses to investigate the relationship between the infiltration abundance of 15 immune cells in the GSE131761 dataset, and the results are presented in Figure 8E. Our findings indicated a positive correlation between the infiltration abundance of these 15 immune cells and that of other immune cells in the dataset.

Correlation analyses were conducted to investigate the associations between the infiltration abundance of 15 immune cells and the expression levels of 10 LMDEGs in the GSE131761 dataset. The results were presented in Figure 8F, with a cutoff of p < 0.05. Our findings demonstrated significant negative correlations (p < 0.05) between the infiltration abundance of the 15 immune cells and the expression of the 10 LMDEGs in the GSE131761 dataset, with *LDHA, FOXM1,* and *CDKN3* exhibiting particularly strong correlations with the infiltration abundance of the 15 immune cells.

### 3.7 Immune characteristic analysis by CIBERSORT

**FIGURE 9:**
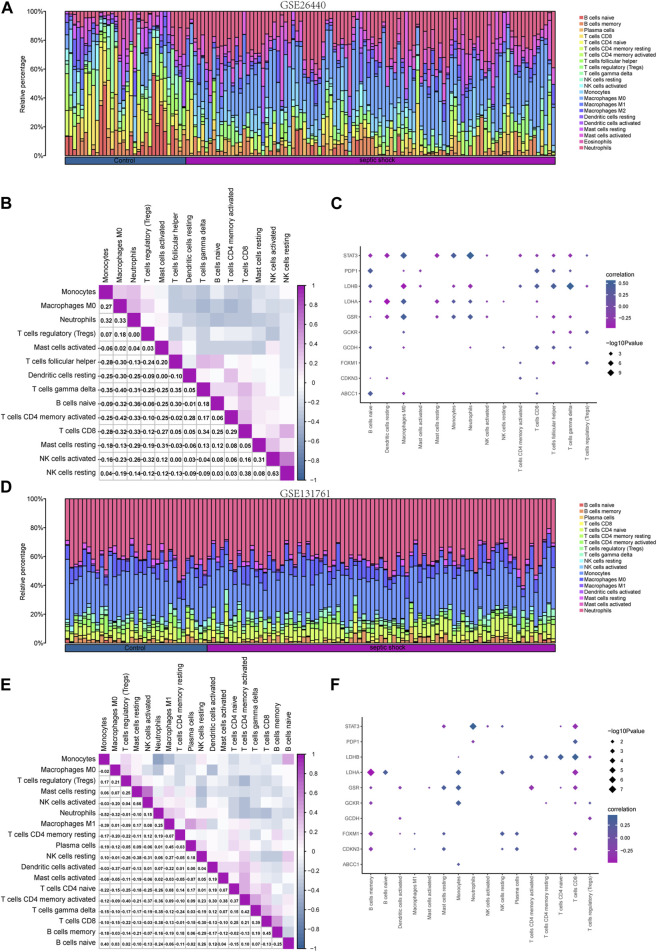
Immune infiltration analysis of the GSE26440 and GSE131761 datasets by CIBERSORT. **(A)** The comparative results of CIBERSORT immune infiltration analysis in GSE26440. **(B)** The correlation analysis results of immune cell infiltration abundance in GSE26440. **(C)** The correlation heat map of immune cells and LMDEGs in GSE26440 dataset. **(D)** The comparative results of CIBERSORT immune infiltration analysis in GSE131761. **(E)** The correlation analysis results of immune cell infiltration abundance in GSE131761. **(F)** The correlation heat map of immune cells and LMDEGs in GSE131761.

To investigate the differences in immune cell infiltration in the GSE26440 dataset, we utilized the CIBERSORT algorithm to calculate the correlations between the expression profiles of 22 immune cells and different groups. Using the immune infiltration analysis results, we generated a stacked bar chart to display the immune cell infiltration levels of each sample in the GSE26440 dataset for the 22 immune cell types (Figure 9A). The findings revealed that 14 immune cell types exhibited statistically significant differences (*p* < 0.05) in infiltration levels between the septic shock and control groups in GSE26440.

Next, we computed the correlation between the infiltration levels of the 14 immune cell types identified in the GSE26440 dataset, and the outcomes were illustrated in Figure 9B. Our findings revealed a negative correlation between the infiltration abundance of most of the 14 immune cell types in the GSE26440 dataset.

Furthermore, we conducted a correlation analysis to investigate the association between the infiltration abundance of the 14 immune cell types in the GSE26440 dataset and the expression levels of 10 LMDEGs. The results were presented in Figure 9C, where a *p*-value threshold of less than 0.05 was used to screen for significant correlations. Our findings revealed significant correlations between the infiltration abundance of the 14 immune cell types and the expression of the 10 LMDEGs in the GSE26440 dataset. As demonstrated, LMDEGs (including STAT3, LDHA, and GSR) showed a negative correlation with NK activated cells.

Using the CIBERSORT algorithm, we conducted an immune infiltration analysis on the GSE131761 dataset. A stacked bar chart displaying the immune cell infiltration levels of 22 immune cells in each sample of the GSE131761 dataset was generated based on the results (Figure 9D). The analysis revealed significant differences in the infiltration levels of 18 immune cells between the septic shock and control groups in the GSE131761 dataset.

Subsequently, the correlations between the infiltration abundance of the 18 immune cells that exhibited infiltration levels greater than zero in the GSE131761 dataset were computed. These results were presented in Figure 9E, indicating that most of the 18 immune cells in the GSE131761 dataset had a negative correlation with immune cell infiltration.

In addition, we assessed the relationship between the infiltration abundance of 18 immune cell types, whose abundance was greater than zero, and the expression levels of 10 LMDEGs in the GSE131761 dataset (Figure 9F). Our analysis revealed significant associations (*p* < 0.05) between the infiltration abundance of 16 immune cell types and the expression levels of the 10 LMDEGs. Notably, T cells CD8 exhibited a noteworthy positive correlation with 6 LMDEGs associated with lactate metabolism. Furthermore, NK resting cells exhibit a positive correlation with several LMDEGs, including STAT3, LDHA, FOXXM1, and CDKN3. Conversely, NK activated cells demonstrate a negative correlation specifically with STAT3. Additionally, LDHB displays a negative correlation with Treg cells in both datasets when analyzed using the CIBERSORT algorithm.

## 4 Discussion

Septic shock has attracted extensive attention and research due to its high mortality rate, and effective therapeutic targets focusing on the inflammatory pathway have yet to be found (van der Poll et al., 2017). Lactate, as a product of glycolysis, not only causes vasodilation and increased permeability, exacerbating microcirculatory ischemia in septic shock, but also affects immune-inflammatory responses, leading to dysregulation. To our knowledge, no bioinformatics studies exploring the relationship between LMDEGs and septic shock can be retrieved. Therefore, to understand the role of lactate in septic shock, this study identified 10 LMDEGs in septic shock patients through two datasets, and performed enrichment analyses of LMDEGs through KEGG and GO. Meanwhile, we also performed GSEA on the two datasets. Next, we constructed PPI, mRNA-miRNA, mRNA-RBP, and mRNA-TF interaction networks of LMDEGs. In addition, we conducted a comparative analysis of the LMDEGs between the septic shock group and the control group, and predicted the diagnostic efficacy of LMDEGs for septic shock. Finally, we analyzed the characteristics of immune infiltration in septic shock and its correlation with LMDEGs using ssGSEA and CIBERSORT algorithms.

The LMDEGs identified in this study, which are related to septic shock, are not only involved in regulating lactate metabolism, but also multiple studies have shown that they are involved in regulating inflammatory immune responses. *LDHA* and *LDHB* are classic genes involved in lactate metabolism, where *LDHA* primarily catalyzes the conversion of pyruvate and NADH into lactate and NAD to enhance ATP supply, while *LDHB* prevents lactate accumulation by catalyzing the reverse reaction (Certo et al., 2021). In terms of immune regulation, LDH can play a modifying role in gene expression in the nucleus of cells, such as by binding to AU-rich elements in RNA encoding GM-CSF to regulate its expression (Chang et al., 2013). *LDHA* can regulate pro-inflammatory cytokines in macrophages (Song et al., 2019), as well as prevent the upregulation of activated T cell nuclear factor in T cells and NK cells, leading to a decrease in IFN-γ production (Brand et al., 2016). Upregulation of *LDHB* expression can partially reverse the inhibitory state of T cells (Decking et al., 2022). Similarly, *STAT3* is also involved in regulating many immune and inflammatory responses, such as promoting the conversion of pro-inflammatory signals to anti-inflammatory signals (Hillmer et al., 2016), regulating the development and function of effector CD8^+^ T cells during acute infection (Sun et al., 2023), regulating the function of ILC2 effectors (Fu et al., 2022), and participating in regulating dendritic cell maturation to modulate self-immunity (Wang et al., 2021). In addition, GSR can reduce lung epithelial cell damage (Hong et al., 2022); *FoxM1* can regulate the development and migration of immune cells (Zheng et al., 2023); and *PDP1* can regulate the activity of the hypoxia-inducible factor 1 (HIF-1) pathway (Karagiota et al., 2023).

According to the KEGG analysis, HIF-1 was found to be the most significantly enriched pathway in LMDEGs. As an important metabolic sensor, HIF-1 participates in the regulation of numerous immune signaling pathways, such as coordinating differentiation between Treg and TH17 cells (Dang et al., 2011), driving transcriptional changes in immune cells in bone marrow and other lymphoid organs (Taylor and Colgan, 2017), and promoting the activation of macrophages and dendritic cells during inflammation (Ivashkiv, 2020). Cytokines are an essential component of the cytokine storm that occurs in septic shock. We performed GSEA analysis on the DEGs of two datasets and found that DEGs were significantly enriched in the *REACTOME INTERLEUKIN 1 FAMILY SIGNALING/IL-1* pathway in both datasets. In septic patients, extensive vasodilation and glycocalyx degradation often occur as a consequence of inflammatory responses involving vascular endothelial cells (Joffre et al., 2020). NF-kB, as a widely expressed transcription factor, plays a crucial role in the inflammatory response of sepsis, with its activity in endothelial cells being mediated by IL-1 (Ye et al., 2008; Raia and Zafrani, 2022). Vascular relaxation primarily relies on three vasodilatory factors produced by endothelial cells: nitric oxide, prostacyclin, and endothelium-derived hyperpolarizing factors (Zhang et al., 2023). However, the regulatory effects of LMDEGs on these vasodilatory factors await further experimental exploration. The extensive family of interleukins is involved in various immune and acute and chronic inflammatory responses. For example, IL-1α and IL-1β can promote inflammatory responses, while IL-37, IL-38, and IL-1Ra have inhibitory effects on inflammatory responses. IL-18 can have both effects depending on the context (Chan and Schroder, 2020). MiRNAs also have a crucial regulatory role in the immune response during the progression of sepsis and the related pathway *WP MIRNAS INVOLVEMENT IN THE IMMUNE RESPONSE IN SEPSIS* was also significantly enriched in the DEGs of both datasets according to GSEA result. In septic patients, upregulation of *miR-221* and *miR-222* often indicates immune paralysis and worsening organ damage (Seeley et al., 2018). The elevation of *miR-210* levels in monocyte-derived cells is significantly associated with the incidence and mortality rate of sepsis (Virga et al., 2021). *miR-142*, as the core of metabolic reprogramming, directly regulates the glycolysis and immunogenic response of dendritic cells (Sun et al., 2019). *miR-127-3p* and *miR-25-3p* can regulate macrophage phenotype and migration and participate in the activation of antioxidant enzymes (Gusar et al., 2022). *MiR-21* expression in bone marrow cells can efficiently balance the metabolic reprogramming that causes the cytokine storm in sepsis with the anti-inflammatory mediators that are responsible for inflammation. *MiR-21* in bone marrow cells can effectively balance the anti-inflammatory mediators and metabolic reprogramming to drive the cytokine storm (De Melo et al., 2021). In this study, we used the miRDB database to predict and construct 148 pairs of mRNA-miRNA interaction relationships for LMDEGs and searched for TFs that bind to LMDEGs through the CHIPBase and hTFtarget databases. Among them, *STAT3* is both an LMDEG and a transcription factor, and has the most interaction relationships with transcription factors. *STAT3* is involved in immune regulation, and its mutation is the basis for the development of hyper-IgE syndrome, an immunodeficiency syndrome (Holland et al., 2007). In addition, *STAT3* is crucial for controlling the expression of autophagy molecules, regulating immune factors, and recruiting immune cells (Zhang et al., 2022).

Sepsis and septic shock, due to their high mortality rates, have received widespread attention regarding their diagnostic and prognostic markers. In this study, ROC curves were plotted for 8 LMDEGs with identical change trends in two datasets, where the AUC of 6 LMDEGs, including *CDKN3, GSR, FOXM1, STAT3, LDHA* and *LDHB*, were greater than 0.7, indicating that LMDEGs have a certain degree of accuracy in predicting the occurrence of septic shock. Previous studies have demonstrated the diagnostic value of *STAT3* in sepsis-induced myocardial disease and sepsis-related respiratory distress syndrome (Zhang et al., 2019; Song et al., 2023). Metabolism-related genes (She et al., 2022), necrosis-related genes (She et al., 2023), immune-related genes (Zheng et al., 2022) common DEGs related to sepsis and metabolic syndrome (Tao et al., 2023), apoptosis-related genes (Wang et al., 2022), inflammation response signature genes (Jiang et al., 2022), and signature genes for septic shock in children (Fan et al., 2022) have all shown some efficacy in the diagnosis and prognostic assessment of sepsis. In addition, metabolites based on metabolomics analysis (Li Y. et al., 2023), m6A regulatory factors (Li F. et al., 2023), and non-coding RNAs such as *miR-147b* (Trung et al., 2021), lncRNA *THAP9-AS1*, and *TSPOAP1-AS1* (Wu et al., 2020) have all been reported as potential diagnostic or prognostic indicators for sepsis or septic shock.

Immune cells play a central role in the dysregulated response of sepsis. Studies have shown that immune cells in the early stages of sepsis often exhibit an overreactive state, which gradually develops into immune tolerance or immune suppression as the disease progresses (Hotchkiss and Karl, 2003; Arts et al., 2017). The overactive immune response in the beginning of sepsis can activate multiple immune cells to produce large amounts of proinflammatory cytokines and chemokine (Galli, 1993). However, as sepsis progresses to the immune suppression phase, various lymphocyte dysfunctions and increased apoptosis can occur (Le Tulzo et al., 2002; Gustave et al., 2018). Previous research has indicated that increased apoptosis of CD4^+^, CD8^+^, and Th17 lymphocytes (Wu et al., 2013), decreased NK cells (Jensen et al., 2018), and decreased B cells (Gustave et al., 2018) are associated with poor prognosis of sepsis, including shock and death. Tregs have been reported to increase in the circulating blood of septic shock patients (Monneret et al., 2003; Gaborit et al., 2021), which is consistent with our findings (Figure 8A). Another study has verified that Tregs can induce immune suppression in septic shock patients (Liu et al., 2022). Immune suppression is an important factor in the progression of sepsis to shock or death, and lactate is further known to regulate the occurrence of immune suppression and inflammatory response in its local environment (Nolt et al., 2018; Luo et al., 2022). Cheng et al. found that blocking metabolic pathways with metformin reduced cytokine production and increased mortality in a mouse model, and their transcriptomic and metabolic profiling analysis of sepsis patients found that the body actively shifts from oxidative phosphorylation to aerobic glycolysis in the immune defense response (Cheng et al., 2016). As mentioned earlier, genes regulating lactate metabolism affect the immune inflammatory response in septic shock through multiple signaling pathways. In this study, the ssGSEA and CIBERSORT algorithm were used to analyze and compare the immune cell infiltration abundance in septic shock and control groups. Significant differences were found in the immune cell infiltration of various immune cells, including CD8 T cells, Tregs, and natural killer cells, in datasets GSE26440 and GSE131761. Moreover, several LMDEGs, such as *STAT3, LDHB, LDHA, PDP1, GSR, FOXM1* and *CDKN3*, were significantly correlated with various immune cells. Therefore, by analyzing the correlation between LMDEGs and immune infiltration in patients with septic shock, it may be possible to provide new targets and directions to block the dysregulated response of sepsis, thereby effectively reducing the incidence and mortality of septic shock.

This study has several limitations that should be acknowledged. Firstly, the lack of animal models and clinical samples hinders the validation of the identified LMDEGs and their correlation with immune infiltration. To address this limitation and enhance the robustness of our findings, future research will focus on incorporating animal models and collecting clinical samples for validation purposes. Additionally, the rapid progression of septic shock and the heterogeneity introduced by differences in specimen collection times may introduce biases in the screening of differentially expressed genes. It is important to consider these factors when interpreting the results and their implications. Moreover, patient metadata, including demographics, genetic background, comorbidities (CCI score), infection type (bacterial, viral, or fungal), and other measures of patient severity (e.g., APACHE II, SOFA scores), hold valuable information. Differential gene analysis based on these diverse metadata can provide a more comprehensive understanding of the mechanisms underlying the occurrence and progression of septic shock. However, due to the study’s design and limitations in the available dataset, it is not possible to conduct analyses incorporating these metadata at the present stage. Future research endeavors should aim to incorporate and analyze these additional patient metadata to gain deeper insights into septic shock.

## 5 Conclusion

Using bioinformatics methods to analyze two datasets of septic shock, we identified 10 lactate metabolism-related genes (*LDHB, STAT3, LDHA, GSR, FOXM1, PDP1, GCDH, GCKR, ABCC1, CDKN3*), and clarified their associated cellular signaling pathways and their relationship with immune cell infiltration, laying the foundation for diagnosis and treatment of septic shock from the perspective of lactate metabolism.

## Data Availability

Publicly available datasets were analyzed in this study. This data can be found here: https://www.ncbi.nlm.nih.gov/geo/query/acc.cgi?acc=GSE26440 https://www.ncbi.nlm.nih.gov/geo/query/acc.cgi?acc=GSE131761.

## Abbreviations

ABCC1, ATP-binding cassette subfamily C member 1; AUC, area under the curve; BP, biological process; CC, cellular component; CDKN3, cyclin-dependent kinase inhibitor 3; DEGs, differentially expressed genes; FOXM1, forkhead box protein M1; GCDH, glutaryl-CoA dehydrogenase; GCKR, glucokinase regulatory protein; GO, Gene ontology; GSEA, gene set enrichment analysis; GSR, glutathione-disulfide reductase; KEGG, Kyoto Encyclopedia of Genes and Genomes; LDHA, lactate dehydrogenase A; LDHB, lactate dehydrogenase B; LMGs, lactate metabolism-related genes; LMDEGs, lactate metabolism-related differentially expressed genes; MF, molecular function; PPI, Protein-protein interaction; RBP, RNA binding protein; PDP1, pyruvate dehyrogenase phosphatase catalytic subunit 1; ROC, Receiver operating characteristic; ssGSEA, single-sample gene-set enrichment analysis; STAT3, signal transducer and activator of transcription 3; TF, transcription factors.
